# The BEACH Domain Protein SPIRRIG Is Essential for Arabidopsis Salt Stress Tolerance and Functions as a Regulator of Transcript Stabilization and Localization

**DOI:** 10.1371/journal.pbio.1002188

**Published:** 2015-07-02

**Authors:** Alexandra Steffens, Andrea Bräutigam, Marc Jakoby, Martin Hülskamp

**Affiliations:** 1 Botanical Institute, Biocenter, Cologne University, Cologne, Germany; 2 Institute of Plant Biochemistry, Cluster of Excellence on Plant Sciences (CEPLAS), Heinrich Heine University, Düsseldorf, Germany; 3 Plant Biochemistry, Heinrich Heine University, Düsseldorf, Germany; University of California Riverside, UNITED STATES

## Abstract

Members of the highly conserved class of BEACH domain containing proteins (BDCPs) have been established as broad facilitators of protein–protein interactions and membrane dynamics in the context of human diseases like albinism, bleeding diathesis, impaired cellular immunity, cancer predisposition, and neurological dysfunctions. Also, the Arabidopsis thaliana BDCP SPIRRIG (SPI) is important for membrane integrity, as *spi* mutants exhibit split vacuoles. In this work, we report a novel molecular function of the BDCP SPI in ribonucleoprotein particle formation. We show that SPI interacts with the P-body core component DECAPPING PROTEIN 1 (DCP1), associates to mRNA processing bodies (P-bodies), and regulates their assembly upon salt stress. The finding that *spi* mutants exhibit salt hypersensitivity suggests that the local function of SPI at P-bodies is of biological relevance. Transcriptome-wide analysis revealed qualitative differences in the salt stress-regulated transcriptional response of Col-0 and *spi*. We show that SPI regulates the salt stress-dependent post-transcriptional stabilization, cytoplasmic agglomeration, and localization to P-bodies of a subset of salt stress-regulated mRNAs. Finally, we show that the PH-BEACH domains of SPI and its human homolog FAN (Factor Associated with Neutral sphingomyelinase activation) interact with DCP1 isoforms from plants, mammals, and yeast, suggesting the evolutionary conservation of an association of BDCPs and P-bodies.

## Abstract 2

During salt stress in the model plant *Arabidopsis thaliana*, the BEACH domain-containing protein SPIRRIG plays a role in the localization of transcripts and formation of mRNA granules, thereby regulating mRNA stability.

## Author Summary

BEACH (*b* *eige* and Chediak Higashi) domain containing proteins (BDCPs) are a highly conserved protein family in eukaryotes. BDCPs are known to be important for membrane dynamics such as vesicle transport, membrane fission and fusion events, and autophagy. Here we describe a new, membrane-independent molecular function of the *Arabidopsis thaliana* BDCP SPIRRIG (SPI) in the regulation of mRNA stability in P-bodies. We report that SPI associates with P-bodies—cytoplasmic mRNA or protein aggregations responsible for the posttranscriptional regulation of RNA storage and decay—in a salt stress-dependent manner. We show that *spi* mutants display salt hypersensitivity suggesting that SPI regulates the uptake of salt stress-regulated mRNAs to P-bodies concomitant with their stability. Finally, we found a direct interaction between the P-body core component DECAPPING PROTEIN1 (DCP1) and SPI and show that this interaction is evolutionarily conserved.

## Introduction

BEACH (*b* *eige* and Chediak Higashi) domain containing proteins (BDCPs) represent a highly conserved protein family in eukaryotes [1,2]. Initially, the BEACH domain was described as a protein motif in the human lysosomal trafficking regulator protein (LYST). Mutations in *LYST* cause the autosomal recessive human Chediak Higashi Syndrome (CHS) [3]. Its corresponding mouse model known as *beige* was characterized in parallel [4,5]. Individuals concerned suffer from severe morphological symptoms, like decreased pigmentation, bleeding diathesis, impaired cellular immunity [6], cancer growth [7], and neurological dysfunctions [8]. To date, all experimental data point to a role of BDCPs in the regulation of membrane dynamics. Mutations in BDCPs have been shown to impair diverse cellular mechanisms, including vesicle transport, membrane fission and fusion events, receptor signaling, autophagy, and apoptosis [9].

In plants, only the BDCP encoding gene *SPIRRIG (SPI)* has been characterized in more detail in the plant model *Arabidopsis thaliana* [2]. Like most BDCPs, SPIRRIG encodes a large protein of 3571aa. Its C-terminally located BEACH domain is preceded by a Pleckstrin-Homology (PH) domain, which is followed by five WD40 repeats. The structural organization of these three domains is highly conserved across all known BDCPs and might function as an independent cassette within these proteins [1,2,10]. Therefore, this region is referred to as the PH-BEACH-WD40 (PBW) module [11]. In the N-terminus, SPI contains multiple ARMADILLO repeats and a Concanavalin A (ConA)-like lectin domain, similarly as the human LYST protein [12]. As mutations in SPI cause defects in epidermal cell expansion and vacuolar integrity, plant BDCPs are also involved in membrane-dependent cellular processes [2]. Our molecular analysis of SPI sheds light on an unexpected, membrane-independent function for a BDCP. We identified the DECAPPING PROTEIN1 (DCP1) as a direct interactor of SPI. DCP1 is known to activate the pyrophosphatase activity of the DECAPPING PROTEIN2 (DCP2), by forming a complex together with VARICOSE (VCS) and the DECAPPING PROTEIN5 (DCP5) [13,14]. Their combined action leads to a 5´monophosphorylated mRNA body, which in turn is accessible for its final decay by the EXORIBONUCLEASE4 (XRN4) [15–21]. Frequently, several of those decapping complexes accumulate with each other. These membrane-unbound ribonucleoprotein (RNP) particles are present in the cytoplasm of all eukaryotic organisms and harbor not only the 5’ to 3’ mRNA decay machinery but also translationally repressed mRNAs, as well as components responsible for mRNA quality control and miRNA-dependent gene silencing. Following the principles of phase transitions, the individual protein constituents and mRNA substrates locally concentrate and form a specific type of microscopically visible RNA granule, termed mRNA processing body (P-body) [22–24]. A general prediction is that P-body formation is directly proportional to the pool of cytoplasmic, translationally repressed mRNAs [25]. However, the molecular mechanisms required for transition of mRNAs from sites of active translation to P-bodies is currently poorly understood.

In this study, we report that the BDCP SPI is a regulator of RNP particle formation in the context of post-transcriptional salt stress response. We show that SPI interacts with DCP1, and that it localizes to P-bodies under salt stress conditions. In addition, salt stress-dependent P-body assembly is impaired in *spi* mutants. Genome-wide transcript analysis by RNA-seq indicated that RNA abundance is pleiotropically altered in *spi* mutants under control and salt stress conditions. Under salt stress, one-third of the transcriptional response is altered in the mutant as compared to wild type. We tested whether the loss of P-body formation in *spi* mutants is indicative for the post-transcriptional regulation of salt stress-regulated transcripts. We found that SPI is required for the stabilization of a subset of salt stress-regulated mRNAs and their recruitment to P-bodies. As *spi* mutants display salt hypersensitivity, it is conceivable that the salt stress-dependent regulative function of SPI at P-bodies is biologically relevant. Finally, we show that an interaction between BDCPs and DCP1 is evolutionarily conserved, suggesting that the role in RNP particle formation is a general feature of eukaryotic BDCPs.

## Results

### SPI Interacts with the P-body Component DCP1

**Fig 1:**
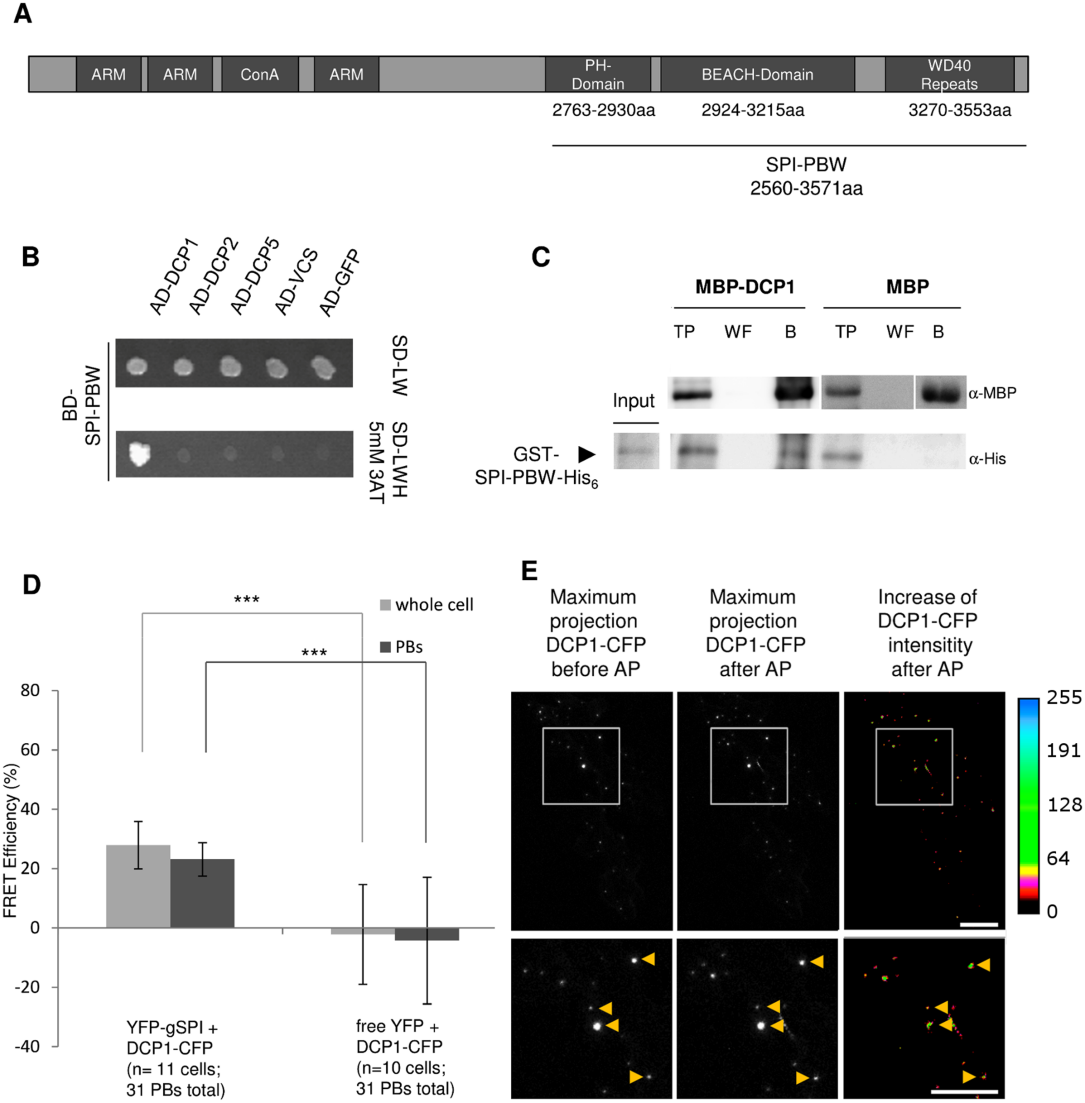
The SPI protein interacts with DCP1. (A) Schematic presentation of the domain organization of the SPI protein: the ARMADILLO repeats (ARM), the Concanavalin A-like lectin domain (ConA), and the C-terminal PBW module (SPI-PBW). (B) Yeast two-hybrid interactions. Upper part: double transformed yeast cells on selective dropout medium lacking leucine (-L) and tryptophan (-W). Bottom part: interaction between SPI-PBW, N-terminally fused to the GAL4 Binding Domain (BD), and DCP1 and other P-body core components fused to the GAL4 Activation Domain (AD), on selective dropout medium lacking leucine (-L), tryptophan (-W), and histidine (-H), supplemented with 5 mM 3-Aminotrizole (3AT). The Green Fluorescent Protein (GFP), N-terminally fused to the GAL4-AD, has been included as negative control. (C) Coprecipitation of SPI-PBW-His_6_ with DCP1-MBP. Throughputs (TP), wash fractions (WF), and resin-bound MBP fusions (B) were detected by α-MBP (upper part) and α-His_6_ (lower part) antibody staining. GST-SPI-PBW-His_6_ (~110 kDa, arrowhead) coprecipitated with MBP-DCP1 (~ 83 kDa), but not with MBP (~42 kDa). Samples detected on different blots are separated by lines. (D) FRET_E_ (in %) was measured in whole leaf epidermis cells (whole cells) and stationary P-bodies (PBs). YFP was bleached in whole cells (for details see Materials and Methods). Mean FRET_E_’s for 35S_pro_:YFP-gSPI and 35S_pro_:DCP1-CFP (n = 11 cells) or 35S_pro_:YFP and 35S_pro_:DCP1-CFP (n = 10 cells) are shown. Error bars represent standard deviations for whole cells, and the standard deviation of the mean for PBs (*n* = 31 stationary PBs derived from whole cell samples). Two-tailed student’s *t* test was performed to compare FRET_E_ between 35S_pro_:YFP-gSPI/35S_pro_:DCP1-CFP and 35S_pro_:YFP/35S_pro_:DCP1-CFP for each group (*** *p* < 0.001). (E) Representative images of 35S_pro_:DCP1-CFP in a transiently transfected leaf epidermis cell prior to (left) and after (middle) Acceptor-photobleaching (AP). For a better visualization, the increase of fluorescence intensity of DCP1-CFP after AP is presented in pseudocolors (right), see color scale for comparison. A group of stationary PBs is highlighted by the boxed area and magnified (lower row). Yellow arrowheads in magnifications mark stationary PBs used for FRET quantifications. Scale bars: 30 μm.

**Fig 2:**
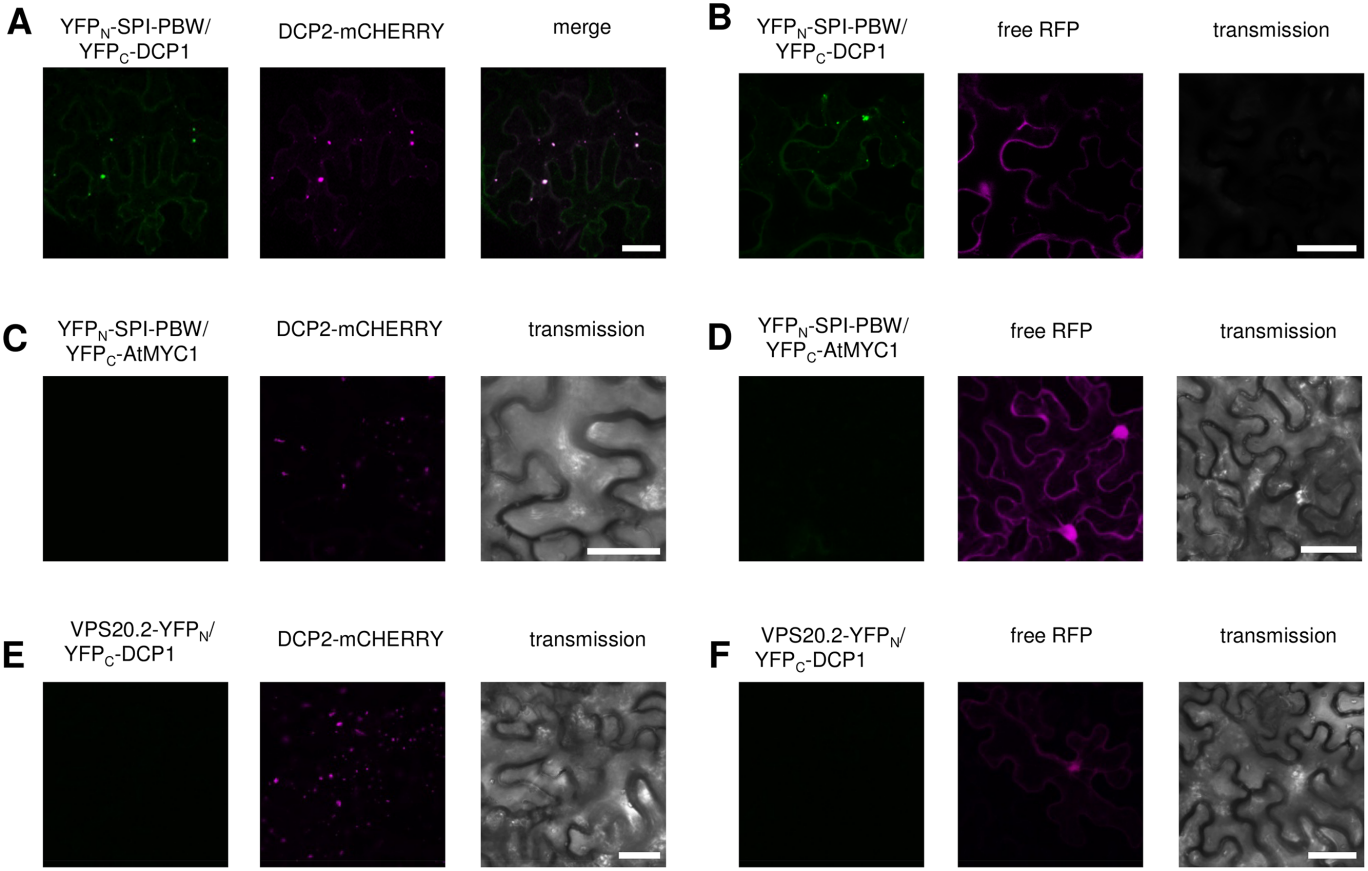
SPI and DCP1 interact at P-bodies in BiFC assays. Fusion proteins are expressed under the control of the 35S promoter. (A) Interaction of YFP_N_-SPI and YFP_C_-DCP1 (left) in transiently transformed *N*. *benthamiana* leaves 72 h post-transfection at DCP2-mCHERRY labeled P-bodies (middle). Right pictures show the overlay of the left (green) and middle (magenta) pictures. (B) Interaction of YFP_N_-SPI and YFP_C_-DCP1 in cytoplasmic dots (left) in *N*. *benthamiana* leaves coexpressing free RFP as transformation control (middle). Right pictures show the corresponding transmission picture. (C–F) Representative images of BiFC negative controls. No YFP signal in leaf epidermis cells coexpressing YFP_N_-SPI-PBW and YFP_C_-AtMYC1 in combination with DCP2-mCHERRY (C) and free RFP (D). No YFP signal in leaf epidermis cells coexpressing YFP_C_-DCP1 and VPS25-YFP_N_ in combination with DCP2-mCHERRY (E) and free RFP (F). Scale bar: 50 μm.

Consistent with a role for BDCPs in membrane trafficking and dynamics, several studies identified membrane-associated proteins as binding partners of BDCPs [26–30]. To identify interactors of plant BDCPs, we performed yeast two-hybrid cDNA library screens using the C-terminal fragment of SPI containing its PBW domain module (referred to as SPI-PBW hereafter, Fig 1A) as bait. Surprisingly, we identified the evolutionarily conserved P-body core component DCP1 as an interaction partner (Fig 1B). All other tested decapping complex proteins including DCP2, DCP5, or VCS did not show interactions with SPI in yeast two-hybrid assays. The interaction of SPI-PBW and DCP1 was confirmed in pull-down experiments with bacterially expressed proteins. Gluthatione S-Transferase (GST)/His_6_-fusions of SPI-PBW were efficiently bound to resins labeled with Maltose Binding Protein (MBP)-tagged DCP1, while no binding was detected with the negative control MBP alone (Fig 1C). To analyze the interaction between full-length SPI and DCP1 in Arabidopsis leaf epidermis cells, we performed Förster-Resonance Energy Transfer (FRET)-Acceptor Photobleaching (AP) experiments (Fig 1D and 1E). We expressed YFP-tagged full-length genomic SPI (acceptor) and DCP1-CFP (cyan fluorescent protein; donor) under the 35S promoter and measured their FRET efficiencies (FRET_E_). We measured FRET_E_ of about 27% in whole cells, indicating that the interaction between DCP1 and SPI occurs in vivo. To test whether the interaction takes place at P-bodies, we analyzed the fraction of immobile P-bodies [31]. Here, the FRET_E_ was 23% (Fig 1E), indicating that SPI and DCP1 interact at P-bodies. No significant FRET was detected between DCP1-CFP and free YFP (yellow fluorescent protein) as a negative control. Donor emissions of cells transfected with DCP1-CFP alone were used as a photobleaching corrective (Fig 1D). The intracellular localization of the SPI-PBW/DCP1 interaction was independently analyzed by Bimolecular Fluorescence Complementation (BiFC) assays in transiently transformed *Nicotiana benthamiana* leaf epidermis cells. Like shown for full-length SPI and DCP1 in FRET-AP assays, we found SPI-PBW and DCP1 interacting at distinct cytoplasmic dot-like structures. These completely colocalized with DCP2-mCHERRY (mCHERRY is a monomeric mutant of DsRED) (Fig 2A). To exclude that the presence of another P-body component influences the interaction behavior of SPI-PBW and DCP1, we confirmed our observations in BiFC assays coexpressing free RFP (red fluorescent protein) instead of DCP2-mCHERRY (Fig 2B).

As negative controls, we coexpressed YFP_C_ (C-terminal half of YFP)-DCP1 and YFP_N_ (N-terminal half of YFP)-SPI-PBW with YFP_N_ C-terminally fused to VPS20.2 (Vacuolar Sorting Protein 20.2) and YFP_C_ N-terminally fused to AtMYC1 (MYC related protein 1), respectively (Fig 2C–2F; S1A and S1B Fig). We did not observe any YFP fluorescence, confirming the specificity of our BiFC analysis (S1 and S2 Tables). The integrity of VPS20.2-YFP_N_ was confirmed in cells cotransfected with its known interactor VPS25 (Vacuolar Sorting Protein 25), C-terminally fused to YFP_C_ (S1C Fig) [32,33]. The integrity of AtMYC-YFP_C_ was confirmed by showing BiFC interaction with GL1 (GLABRA1), C-terminally fused to YFP_N_ (S1D Fig) [34].

Taken together, these data show that Arabidopsis SPI interacts with DCP1 and that this interaction occurs at P-bodies.

### Subcellular Localization of SPI

**Fig 3:**
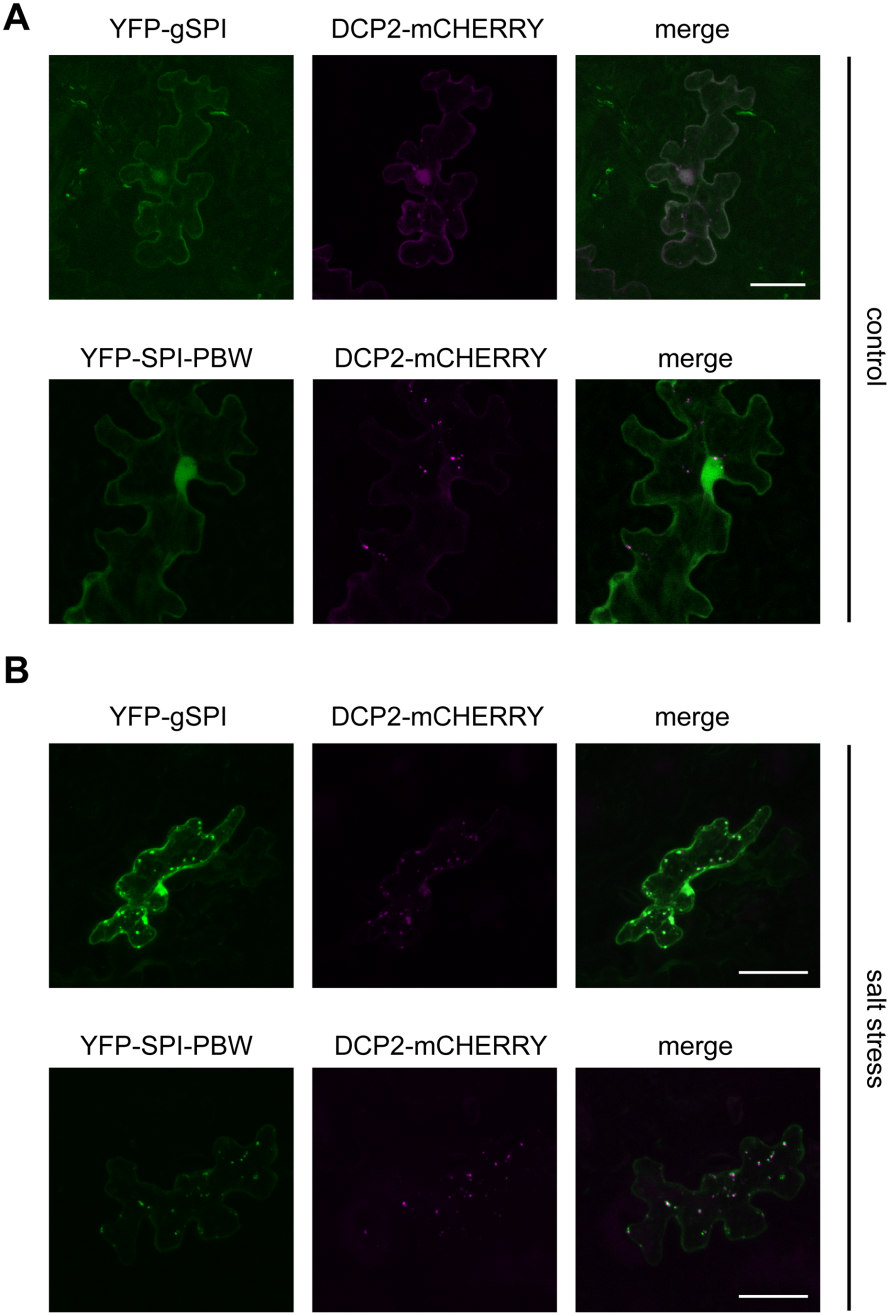
Colocalization studies with YFP-fusions of SPI (column I) and DCP2-mCHERRY-labeled P-bodies (column II) in transiently transfected Arabidopsis leaf epidermis cells. Column III presents the overlay picture between columns I (green) and II (magenta). (A) Under nonstress conditions, YFP-gSPI (upper row) and YFP-SPI-PBW (lower row) are evenly distributed throughout the cytoplasm. (B) YFP-gSPI (upper row) and YFP-SPI-PBW (lower row) accumulate at DCP2-mCHERRY-labeled P-bodies after incubation of transfected leaves on ½Murashige and Skoog (MS) medium supplemented with 140 mM NaCl for 10 h. Scale bar: 25 μm.

The finding that SPI-PBW interacts with DCP1 at P-bodies raised the question of where the SPI protein is localized. In contrast to our expectations from FRET and BiFC assays, we found 35S promoter-driven N-terminal YFP fusions with full-size genomic SPI (35S_pro_:gSPI) as well as SPI-PBW evenly distributed in the cytoplasm (Fig 3A). In less than 10% of cells analyzed, YFP-gSPI accumulated in cytoplasmic dot-like structures (S1E Fig). We did not notice a correlation between the expression strength of YFP-gSPI and its localization behavior. In cells cotransfected with fluorescently tagged DCP1 and YFP-gSPI, YFP-gSPI was efficiently relocalized to P-bodies (84.8%, S1E and S1F Fig). This relocalization behavior suggests that DCP1 can recruit SPI to P-bodies. However, the functional role of DCP1 in the recruitment of SPI to P-bodies could not be analyzed, as *dcp1* mutants are embryonic lethal [35].

### SPI Localizes to P-bodies in a Salt Stress-Dependent Manner

As not only the accumulation frequency of the decapping complex but also the localization of various mRNA and protein constituents to P-bodies is highly stress-regulated [36–39], we reasoned that the association of SPI with P-bodies might be triggered by stress. Different abiotic stress conditions, including salt stress or hypoxia, are known to induce repression of de novo protein synthesis in Arabidopsis [40,41]. In turn, polysomes disassemble and release their transcripts, which are trapped by RNA binding proteins that frequently aggregate into cellular RNP particles such as P-bodies [42]. Consequently, P-bodies increase in number and size [24,38,43–45]. As P-body number increases under salt stress conditions in *A*. *thaliana* (Table 1), we tested whether salt treatments can induce the recruitment of SPI to P-bodies.

**Table 1:** P-body number in leaf epidermis cells transiently expressing DCP1-mCHERRY. Average numbers of P-bodies are provided for Col-0, *spi-2* and *spi-4* under nonstress (½MS) and salt stress (½MS supplemented with 140mM NaCl for 10 h) conditions. Each biological replicate (n) comprises at least 30 cells. SD values represent standard deviations. Two-tailed student’s *t* tests were performed to compare stress and nonstress conditions.

Genotype	Average number of P-bodies (1/2MS)	Average number of P-bodies (1/2MS +140mM NaCl)	Fold-change relative to nonstress conditions (1/2MS)
Col-0	11.5	18.5	1.8 (*)
(n = 8)	(SD +/- 5.7)	(SD +/- 8.9)	(SD +/- 0.6)
*spi-4*	10.4	12.47	1.2
(n = 7)	(SD +/- 2.2)	(SD +/- 1.9)	(SD +/- 0.2)
*spi-2*	11.6	14.5	1.3
(n = 8)	(SD +/- 4.5)	(SD +/- 6.3)	(SD +/- 0.5)

* = *p* < 0.05

In transiently transfected leaves, incubated on ½ Murashige and Skoog (MS) control medium or ½MS medium supplemented with different NaCl concentrations (S1G Fig), we observed a salt-dependent accumulation of YFP-gSPI in cytoplasmic dots that colocalized with the P-body marker DCP2-mCHERRY (Fig 3B). In contrast, Mannitol treatments had no effect on the localization of YFP-gSPI indicating that the relocalization of SPI is not triggered by osmotic stress in general (S1H Fig). The salt stress-dependent localization of SPI to P-bodies is likely mediated by its PBW module, as the corresponding fragment alone was sufficient for the localization to P-bodies (Fig 3A and 3B, second rows). Free YFP was never observed to accumulate in dots (S1I Fig).

In summary, these data show that the localization of SPI to P-bodies is triggered by salt stress but not by osmotic stress in general.

### Salt Stress Induced P-body Formation Depends on SPI

**Fig 4:**
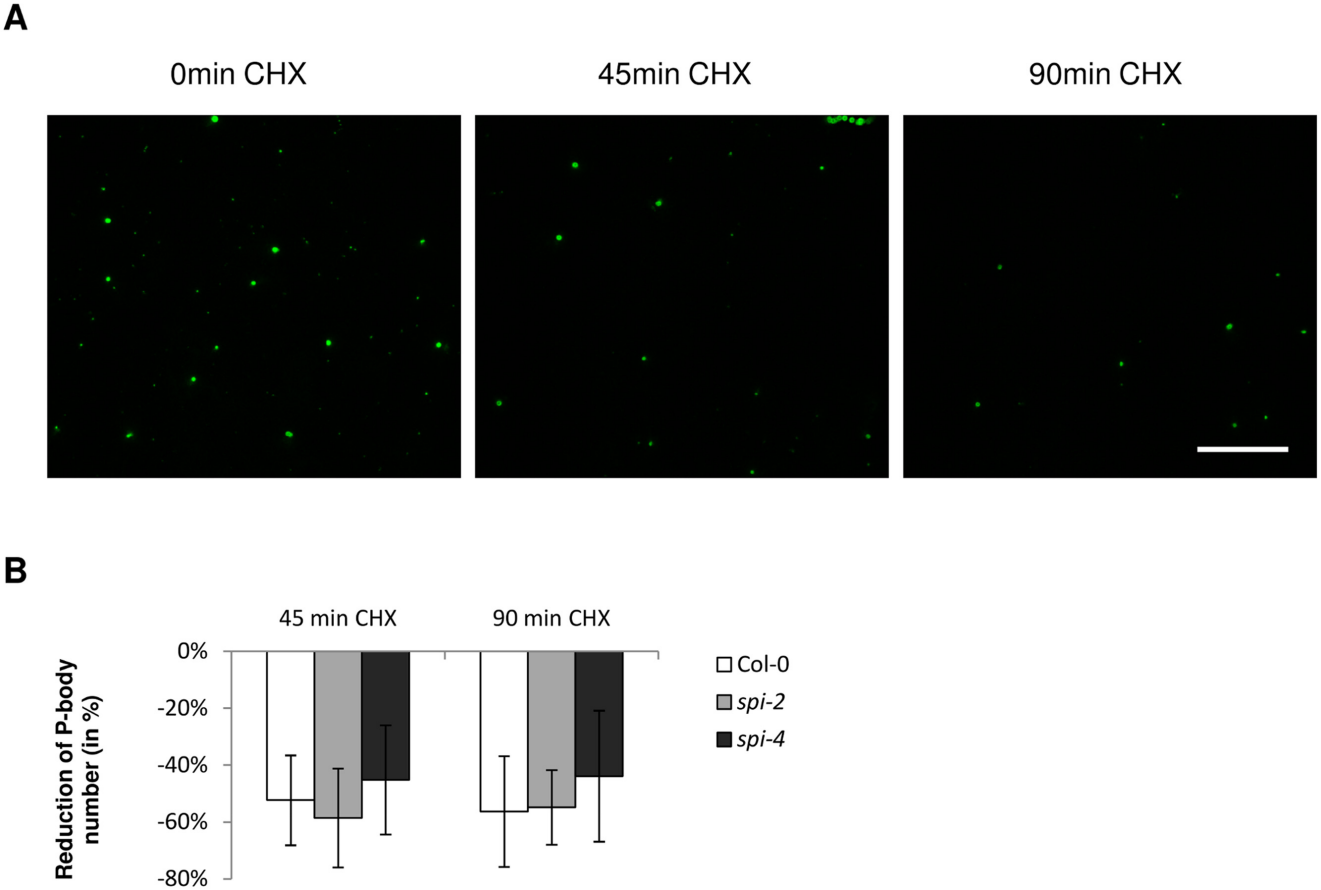
Blocking translation leads to a reduction of P-body number. Changes of P-body number were analyzed in whole leaf areas of transgenic plants expressing DCP1-YFP. (A) Whole leaf areas are presented before (0 min) and after continuous CHX treatments (45 min and 90 min). Scale bar: 50 μm. (B) Reduction of P-body number (in %) relative to untreated samples (time point 0) determined 45 min and 90 min after CHX treatment. Data denote the average from seven biological replicates. SD values represent standard deviations. No statistical differences were found between wild-type and *spi* mutants (Two-tailed student’s *t* test).

P-body formation is determined by the relative entry and exit rates of mRNAs and mRNA binding proteins. The efficiency of material uptake and release from P-bodies can be monitored after blocking translation elongation with Cycloheximide (CHX), which causes the trapping of mRNAs in polysomes. As a consequence, the pool of translationally repressed mRNAs shrinks and the influx into P-bodies decreases, resulting in a reduced number of P-bodies [14,24,44–46]. In stably transformed 35S:DCP1-YFP Col-0 plants, the number of P-bodies was reduced by 50%, 45 min after CHX treatment (Fig 4A and 4B). The reduction of P-body numbers was not significantly different in two different *spi* mutant alleles (S2A and S2B Fig), indicating that uptake and release from P-bodies are not generally affected in *spi* mutants under nonstress conditions. Mock treated cells did not show any significant changes in P-body numbers (S2C Fig). We confirmed the response of P-bodies to CHX treatments in leaf epidermis cells of Col-0, *spi-2*, and *spi-4* transiently transfected with another P-body marker, 35S_pro_:DCP2-mCHERRY (S2D Fig).

To test whether the DCP1-dependent recruitment of SPI requires the bulk RNA flow from polysomes to P-bodies, we exposed transiently transfected cells to 0.5 mM CHX for 150 min. Under these conditions, YFP-gSPI still accumulated in DCP1-mCHERRY labeled P-bodies (S1G and S2E Figs).

In a second step, we examined P-body assembly under salt stress conditions in two *spi* alleles. In contrast to wild-type, both *spi* mutants showed no significant changes in P-body number (Table 1). In samples treated simultaneously with CHX (0.5 mM) and NaCl (140 mM) for 90 min, the number of P-bodies decreased significantly by 20% in both Col-0 and *spi* mutants (S2F Fig). This indicates that in Col-0, the salt stress-induced increase of P-body number depends on the RNA flow from polysomes to P-bodies. In addition, the data show that in *spi* mutants P-body maintenance depends on polysome-P-body RNA shuttling also under salt stress conditions.

Taken together, our data suggest that SPI is functionally important for the accumulation of mRNA-protein complexes in P-bodies under salt stress conditions.

### *spi* Mutants Display Salt Hypersensitivity

**Fig 5:**
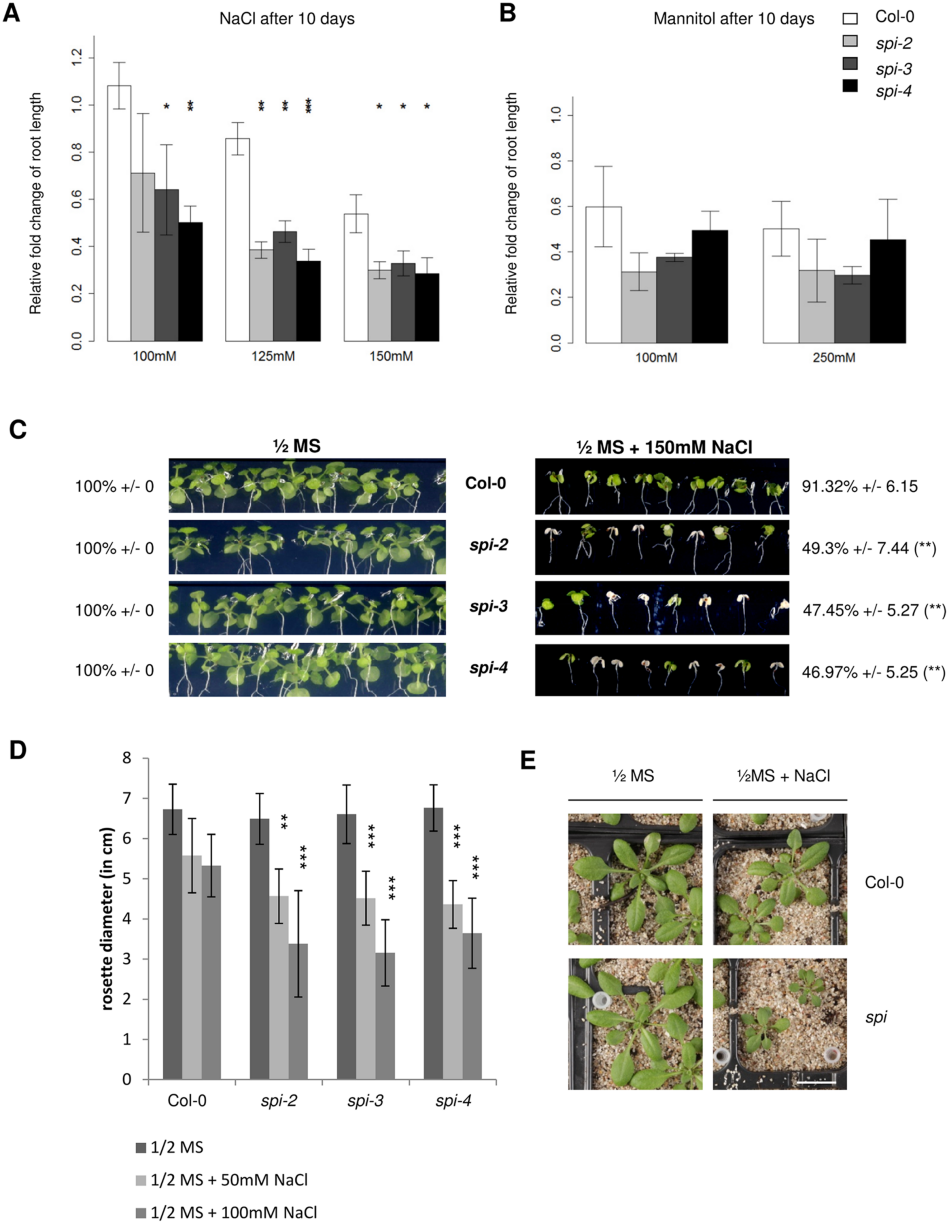
*spi* mutants display salt hypersensitivity. Relative changes of root length after 10 d on ½MS plates supplemented with (A) 100 mM, 125 mM, and 150 mM NaCl or (B) 100 mM and 250 mM Mannitol (Man). Data in (A) and (B) were normalized to nonstress conditions and denote the average from three independent biological replicates (*n* = 12 seedlings each). Error bars represent standard deviations. Two-tailed student’s *t* tests were performed to compare *spi* alleles and Col-0 exposed to the same conditions (* *p* < 0.05; ** *p* < 0.01; *** *p* < 0.001). (C) Cotyledon greening of seedlings measured after 14 d on ½MS plates supplemented with 150 mM NaCl. Greening efficiencies (in %) denote the average from three independent biological replicates (*n* = 12 seedlings each). Errors represent standard deviations. Two-tailed student’s *t* tests were performed to compare *spi* alleles and Col-0 exposed to the same conditions (* *p* < 0.05; ** *p* < 0.01; *** *p* < 0.001). (D) Diameter of leaf rosettes (in cm) of 32-day-old plants, measured after irrigation with ½MS only (control) or ½MS supplemented with increasing NaCl concentrations on every second day (two times ½MS + 50 mM NaCl and two times ½MS +100 mM NaCl). Data denote the average from three biological replicates (*n* = 14 plants each). Error bars represent standard deviations. Two-tailed student’s *t* tests were performed to compare *spi* alleles and Col-0 exposed to the same conditions (* *p* < 0.05; ** *p* < 0.01; *** *p* < 0.001). (E) Representative images of 30-d-old Col-0 and *spi-2* plants grown under nonstress (½MS) and salt stress conditions (irrigation two times with ½MS + 50 mM NaCl and one time with ½MS + 100 mM NaCl in alternation with ½MS every second day) on a sand–soil mixture. Scale bar: 1.5 cm.

The salt stress-dependent function of SPI at P-bodies suggested that SPI might be relevant for the salt stress tolerance of Arabidopsis. We first compared root growth efficiencies between Col-0 and three *spi* mutant alleles at different NaCl concentrations under nontranspiring conditions. The relative growth of primary roots did not differ between wild-type and *spi* under nonstress conditions. With increasing NaCl concentrations, we observed a stronger inhibition of primary root growth in *spi* than in Col-0 (Fig 5A). The stress hypersensitive phenotype of *spi* mutants is salt specific, as the relative root growth of *spi* and Col-0 did not significantly differ after Mannitol treatments (Fig 5B). The notion that *spi* mutants are salt hypersensitive was supported by cotyledon greening assays. While cotyledon greening of *spi* mutants was undistinguishable from Col-0 plants under nonstress conditions, a clear whitening of more than 50% of *spi* seedlings was observed on MS medium supplemented with 150 mM NaCl after an incubation time of 14 d. More than 90% of Col-0 plants remained unaffected under these conditions (Fig 5C). Next, we assessed the salt sensitivity of more adult plants under transpiring conditions in NaCl irrigation experiments (Fig 5D and 5E). When treated with 50 mM or 100 mM NaCl, growth of *spi* mutants was much more restricted than growth of wild type plants.

In summary, these data show that SPI is required for Arabidopsis salt stress but not for osmotic stress tolerance in general.

### The Loss of SPI Leads to Pleiotropic Transcriptional Changes

To test whether the hypersensitive response of *spi* mutants is accompanied by changes in the transcript levels, we performed a genome-wide analysis of the transcriptome by RNAseq of wild type and *spi* mutants under control and salt stress conditions. 10-d-old seedlings were incubated in liquid ½MS control medium or ½MS medium supplemented with 200 mM NaCl for 4 h. Ten libraries were Illumina-sequenced, mapped, and the results were visualized (for additional information see S1 Text, S3–S8 Figs, and S3 Table; complete raw data are available under http://www.ncbi.nlm.nih.gov/bioproject/278120). Between *spi* mutant and wild type, 483 (control condition) and 474 transcripts (salt-treated condition) were significantly different between genotypes (q < 0.01, Benjamini-Hochberg (BH) corrected). The salt treatment significantly altered the abundance of 8,469 transcripts in Col-0 and 8,482 transcripts in the *spi* mutant (Table 2).

**Table 2:** Significantly changed gene expression between Col-0 and *spi*. Data present only those with a q-value < 0.01, Benjamini Hochberg (related to Fig 6).

	Col-0/*spi*	Col-0/Col-0 NaCl	*spi*/*spi* NaCl	Col-0 NaCl/ *spi* NaCl
**significantly down**	**145**	**3770**	**3992**	**258**
**significantly up**	**338**	**4699**	**4490**	**216**

**Fig 6:**
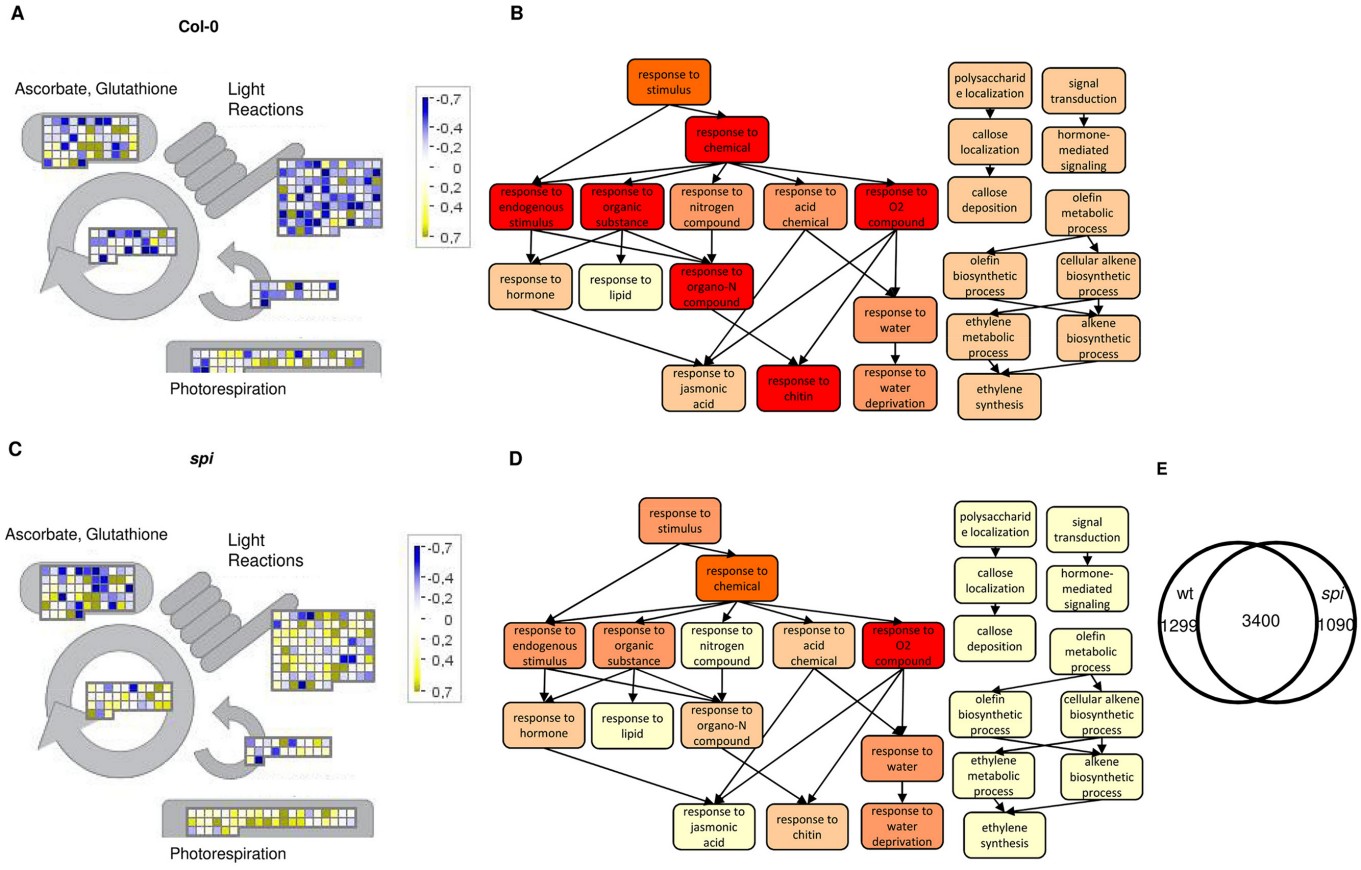
Transcriptome-wide analysis. (A) Mapman visualization of log2-fold changes in Col-0 upon salt treatment and (B) the corresponding 25 most strongly enriched GO terms. Darker colors in GO term categories represent higher q-values (BH-corrected). (C) Mapman visualization of log2-fold changes in *spi* upon salt stress induction and (D) the corresponding 25 most strongly enriched GO terms in *spi*. For color codes see 6C. (E) Venn diagram comparing the salt stress-dependent up-regulation of transcripts in Col-0 (wt) and *spi* (see also S8 Table).

In Col-0, the salt treatment altered transcriptional abundance in stress-related gene categories (Fig 6A and 6B). At the same time, transcript abundance in categories related to growth was changed (S5 and S6 Figs). This transcript abundance pattern reflected the cross activation of different stress pathways, the preparation for reduced nutrient uptake with regard to nitrate and iron, and, likely as a consequence, a down-regulation of photosynthesis (Fig 6A). In *spi*, the salt treatment altered transcriptional abundance in similar gene categories, including the main abiotic responses, biotic responses, and metabolic Gene Ontology (GO) term categories (Fig 6C and 6D, S7–S9 Figs). The gene-by-gene comparison of the transcriptional abundance changes in Col-0, and *spi* showed that one-fourth of the responses are specific to each genotype and three-fourths are shared (Fig 6E). In the shared response, the strength of regulation was similar between Col-0 and *spi*, indicating that the response to salt stress is not attenuated but qualitatively changed (S7–S9 Figs). As the biological relevance for the salt stress response has not been validated for most of the differentially regulated genes yet, we directly compared the expression levels of a subset of genes, functionally and/or genetically shown to regulate Arabidopsis salt stress response [47–53]. While most of the candidate genes tested were significantly up-regulated under salt stress conditions in Col-0 as well as in *spi* mutants, a subset of genes was either not up-regulated or significantly less up-regulated in *spi* mutants (S4 Table).

Our RNA-Seq analysis demonstrated that SPI affects transcripts abundance pleiotropically in a global manner rather than specifically salt stress-regulated mRNAs. Among the latter, only a subset of transcripts was identified to be differentially regulated by SPI.

### Transcripts Are Destabilized under Salt-Stress Conditions in *spi*

**Fig 7:**
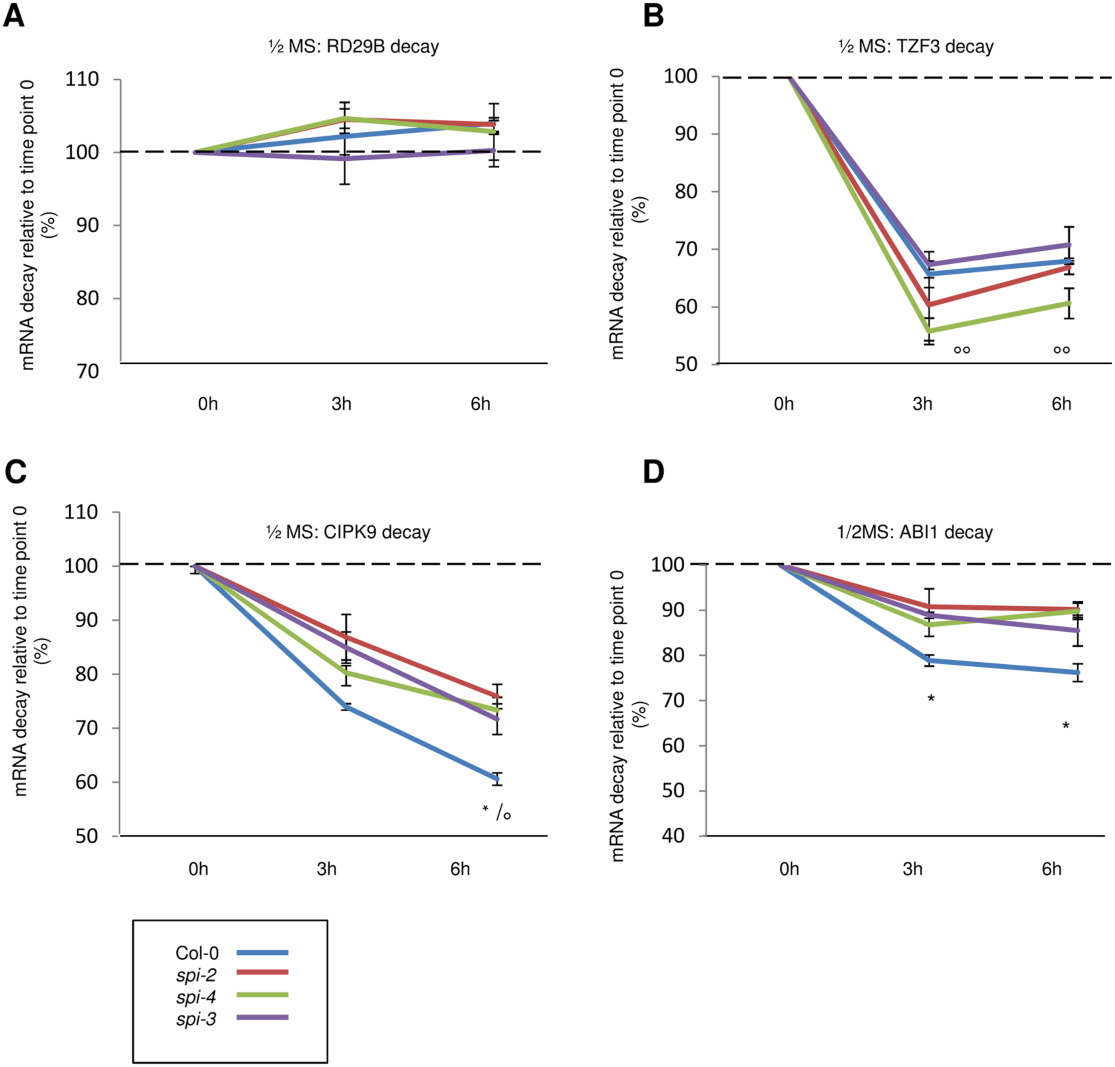
mRNA decay is not accelerated in *spi* mutants under nonstress conditions. mRNA stabilities (in %) were determined 3 h and 6 h after application of Actinomycin D (ActD), relative to time point 0 under nonstress conditions for RD29B (A), TZF3 (B), CIPK9 (C), and ABI1 (D). Data denote the average from three independent biological and two technical replicates. Error bars represent the standard error of the mean. Two-tailed student’s *t* tests were performed to compare 18S rRNA normalized expression levels either of *spi* alleles with that of wild-type (* *p* < 0.05; ** *p* < 0.01 of all *spi* alleles), or of ActD-treated samples with that of untreated samples (time point 0) (° *p* < 0.05; **°°** *p* < 0.01 of all treated samples).

Since the steady-state transcript levels provide only a snapshot of the balance between transcription and mRNA decay, we determined the stabilities of SPI-dependent (TANDEM ZINC FINGER PROTEIN 3 (TZF3) and ABA INSENSITIVE 1 (ABI1)) and SPI-independent regulated mRNAs (RESPONSIVE TO DESSICATION 29B (RD28B) and CBL-INTERACTING PROTEIN KINASE 9 (CIPK9)) (S4 Table). Towards this end, we measured transcript levels by qPCR under nonstress and salt stress conditions 3 h and 6 h after inhibition of transcription with Actinomycin D (ActD) [54,55]. Under nonstress conditions, the mRNA decay rates of RD29B and TZF3 were similar in wild type and *spi* mutants (Fig 7A and 7B), while ABI1 and CIPK9 transcripts were significantly stabilized in all three *spi* alleles (Fig 7C and 7D).

**Fig 8:**
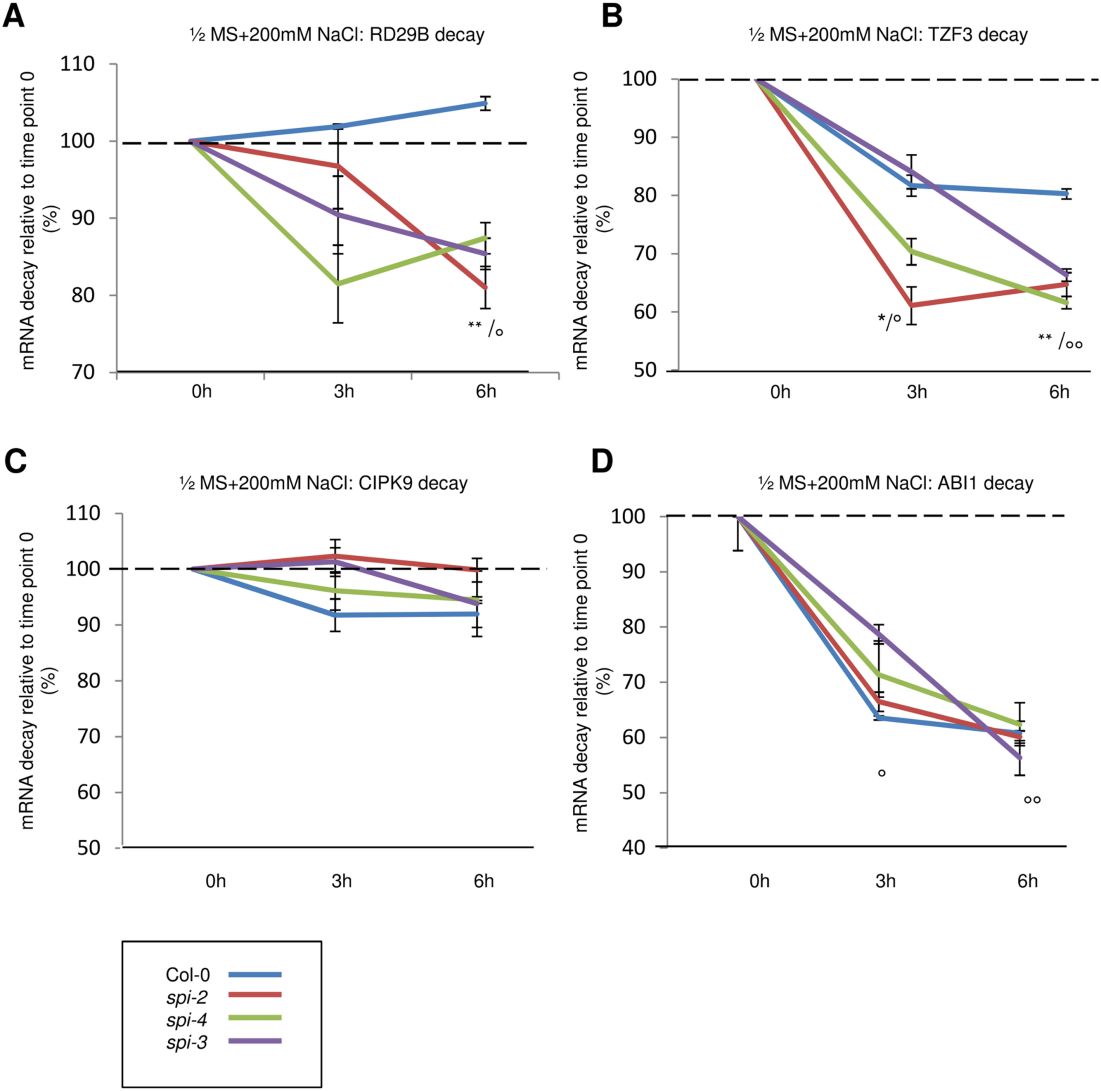
Role of SPI in the regulation of mRNA stability. mRNA stabilities (in %) were determined 3 h and 6 h after application of Actinomycin D (ActD) relative to time point 0 under salt stress conditions (200 mM NaCl in ½MS liquid medium for 4 h). mRNA decay was determined for RD29B (A), TZF3 (B), CIPK9 (C), and ABI1 (D). Data denote the average from three independent biological and two technical replicates. Error bars represent the standard error of the mean. Two-tailed student’s *t* tests were performed to compare 18S rRNA normalized expression levels either of *spi* alleles with that of wild-type (* *p* < 0.05; ** *p* < 0.01 of all *spi* alleles), or of ActD-treated samples with that of untreated samples (time point 0) (° *p* < 0.05; **°°** *p* < 0.01 of all treated samples).

The incubation of seedlings in ½MS liquid medium supplemented with 200 mM NaCl for 4 h resulted in a stabilization of RD29B in wild type. In contrast, RD29B mRNA decay was enhanced in *spi* mutants under these conditions (compare Figs 7A and 8A). Similarly, TZF3 was slightly stabilized under salt stress conditions in wild type (compare Figs 7B and 8B), whereas it was significantly destabilized in the absence of SPI (Fig 8B). CIPK9 and ABI1 mRNA stability was not significantly different in wild type and *spi* mutants (Fig 8C and 8D).

Given that in *spi* mutants some mRNAs are stabilized under control conditions (CIPK9, ABI1) whereas others are destabilized under salt stress conditions (RD29B, TZF3), it is conceivable that SPI does not regulate RNA stability directly but rather the uptake into P-bodies where each mRNA experiences its specific fate.

### SPI Regulates the Localization of Its mRNA Targets

The salt stress-dependent recruitment of SPI to P-bodies suggested to us that SPI might regulate the localization of mRNAs. As salt-regulated transcripts were selectively destabilized under salt stress conditions in *spi* mutants, we tested whether their spatial and temporal distributions were also impaired. We monitored the localization of two different mRNA targets of SPI in vivo using the LambdaN22 reporter system [56]. We placed 16 BoxB repeats N-terminally to the 5’ UTR of full-size genomic TZF3 (gTZF3) and RD29B (gRD29B), and coexpressed them with the LambdaN22 protein fused to mVENUS in Arabidopsis leaf epidermis cells. Binding of LambdaN22-mVENUS to the BoxB repeats enables the indirect visualization of the RNAs of interest. Three different negative control experiments were performed: first, the LambdaN22-mVENUS reporter was coexpressed with the BoxB repeats without the mRNA target (S10A Fig). Second, the full-size genomic target constructs without the stem loops but with an N-terminally fused mCHERRY were coexpressed with the reporter construct (S10B and S10C Fig). Third, the LambdaN22-mVENUS reporter was coexpressed with full-size genomic ABF3 (gABF3), a transcript unaffected in its stability in *spi* mutants under all conditions investigated (S10D–S10F Fig). Under all three control conditions, the LambdaN22-mVENUS reporter was detected almost exclusively in the nucleus or in both the nucleus and the cytoplasm.

**Fig 9:**
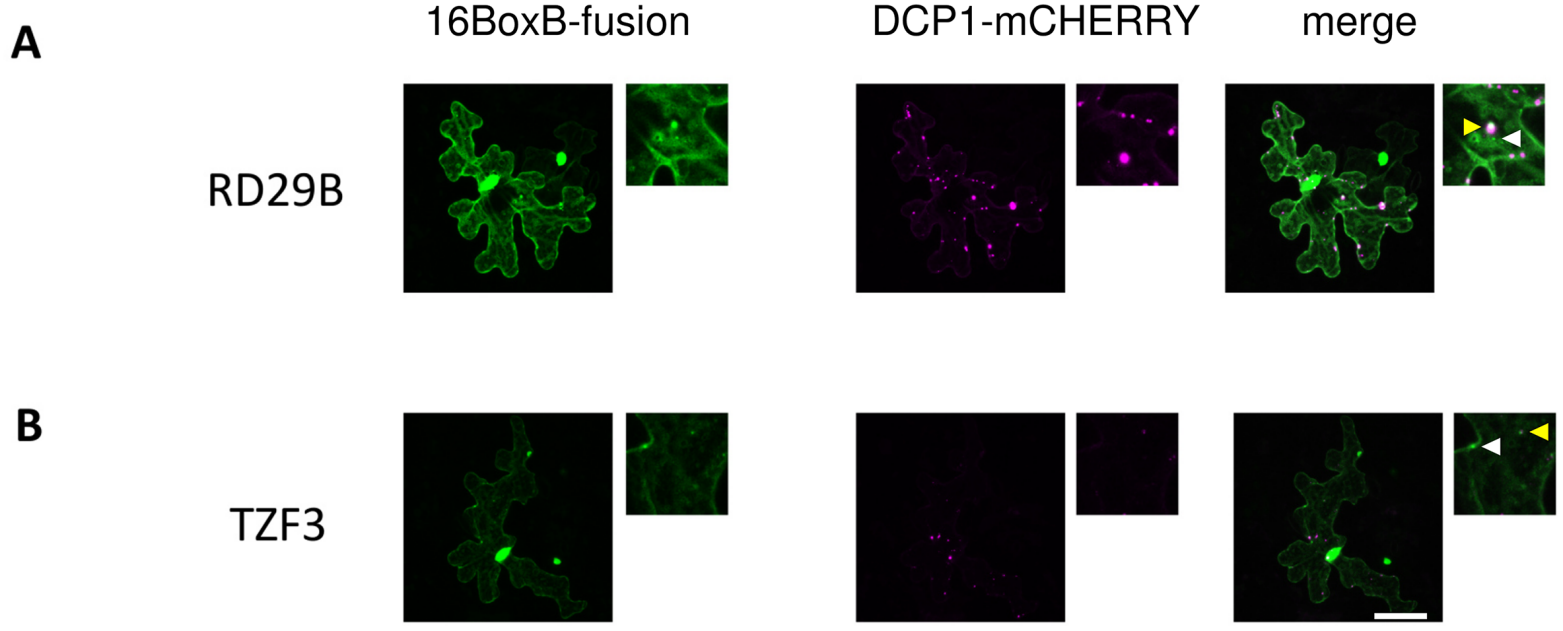
mRNAs of RD29B and TZF3 form cytoplasmic granules. 16BoxB-gRD29B (A) and 16BoxB-gTZF3 (B) are indirectly visualized by the LambdaN22-VENUS reporter in transiently transfected leaf epidermis cells (related to Tables 3 and 4). Column I presents the LambdaN22-mVENUS reporter, column II DCP1-mCHERRY, column III the overlay of column I (green) and II (magenta). Rectangular image magnifications present P-bodies nonoverlapping (white arrowheads) and P-bodies overlapping (yellow arrowheads) RNA accumulations. Scale bar: 25 μm.

In cells coexpressing 16BoxB-gTZF3 or 16BoxB-gRD29, we observed accumulations of the reporter constructs in cytoplasmic dot-like structures, indicating that these accumulations are caused by the presence of the 16BoxB-fused target mRNAs (S11A and S11B Fig). Colocalization studies with DCP1, C-terminally fused to mCHERRY, revealed that several mRNA positive accumulations overlap with P-bodies (Fig 9). As P-body and mRNA granule formation follow the principles of classical liquid–liquid phase separations, comprising multivalent and low affinity interactions between proteins constituents and mRNA substrates involved [57–59], their assembly is highly dynamic. As a consequence, the number of mRNA granules and their size is highly variable from cell to cell [39,46,60,61]. This was also observed in our study; however, a quantitative analysis revealed clear differences between nontreated and salt stress-treated samples. In transfected Col-0 cells, 33% of RD29B and 52% of TZF3 positive dots colocalized with P-bodies under nonstress conditions. Their amount in cells treated with 140mM NaCl increased to 57% and 81%, respectively (Table 3).

**Table 3:** mRNA granules localize to P-bodies (related to Fig 9). mRNA accumulations overlap with P-bodies under nonstress (-NaCl) and salt stress (+NaCl) conditions. Data denote the average (in %) of 65 cells (RD29B) or 120 cells (TZF3).

	RD29B mRNA granules		TZF3 mRNA granules	
	-NaCl	+NaCl	-NaCl	+NaCl
**Col-0**	33.3 (SD +/- 57.1)	57.3 (*) (SD +/-31.7)	56.2 (SD +/- 37.2)	81 (*) (SD +/- 30.1)
***spi-2***	25.9 (SD +/- 20.4)	19.9 (SD +/- 18.4)	45.9 (SD +/- 32.7)	63.9 (SD +/- 34.3)
***spi-4***	49.1 (SD +/- 48.3)	12.5 (SD +/- 17.6)	62.7 (SD +/- 41.1)	45.9 (SD +/- 32.7)

Asterisks represent statistically significant changes in comparison to nonstress conditions (Wilcoxon test; *p* < 0.05).

This salt stress-dependent recruitment of RD29B and TZF3 to P-bodies was not observed after CHX treatments suggesting that these two mRNAs are delivered from the polysomes to the P-bodies (S11C and S11D Fig). Additionally, the total amount of RD29B positive mRNA dots increased drastically in comparison to untreated cells under salt stress conditions (Table 4).

**Table 4:** Numbers of mRNA granules per cell (related to Fig 9). Average number of RD29B (*n* = 65 cells) and TZF3 (*n* = 120 cells) mRNA granules under non stress (-NaCl) and salt stress (+NaCl) conditions. SD values represent standard deviations.

	RD29B mRNA granules		TZF3 mRNA granules	
	-NaCl	+NaCl	-NaCl	+NaCl
**Col-0**	0.16 (SD +/- 0.1)	8.6 (*) (SD +/- 2.2)	1.7 (SD +/-1.6)	0.7 (SD +/- 0.5)
***spi-2***	0.1 (SD +/- 0.1)	0.3 (SD +/- 0.3)	0.07 (SD +/- 0.08)	1.8 (SD +/-1.1)
***spi-4***	0.2 (SD +/- 0.2)	0.15 (SD +/- 0.15)	0.3 (SD +/- 0.17)	1.4 (SD +/- 0.3)

Asterisk represents statistically significant changes in comparison to non stress conditions (Wilcoxon test; p < 0.05).

The average number of TZF3 mRNA dots per cell was unchanged under salt stress conditions in comparison to nonstress conditions (Table 4). In two *spi* alleles, we observed an impaired granule formation behavior for the transcripts of TZF3 and RD29B in two respects. First, no salt stress-dependent relocalization of TZF3 and RD29B mRNA dots to P-bodies took place (Table 3). Second, the total number of RD29B mRNA dots was not increased like in Col-0 cells (Table 4).

Our RNA localization data show that SPI functions as a positive regulator of post-transcriptional RNP particle formation under salt stress conditions.

### The Interaction between BDCPs and P-bodies Is Evolutionarily Conserved

**Fig 10:**
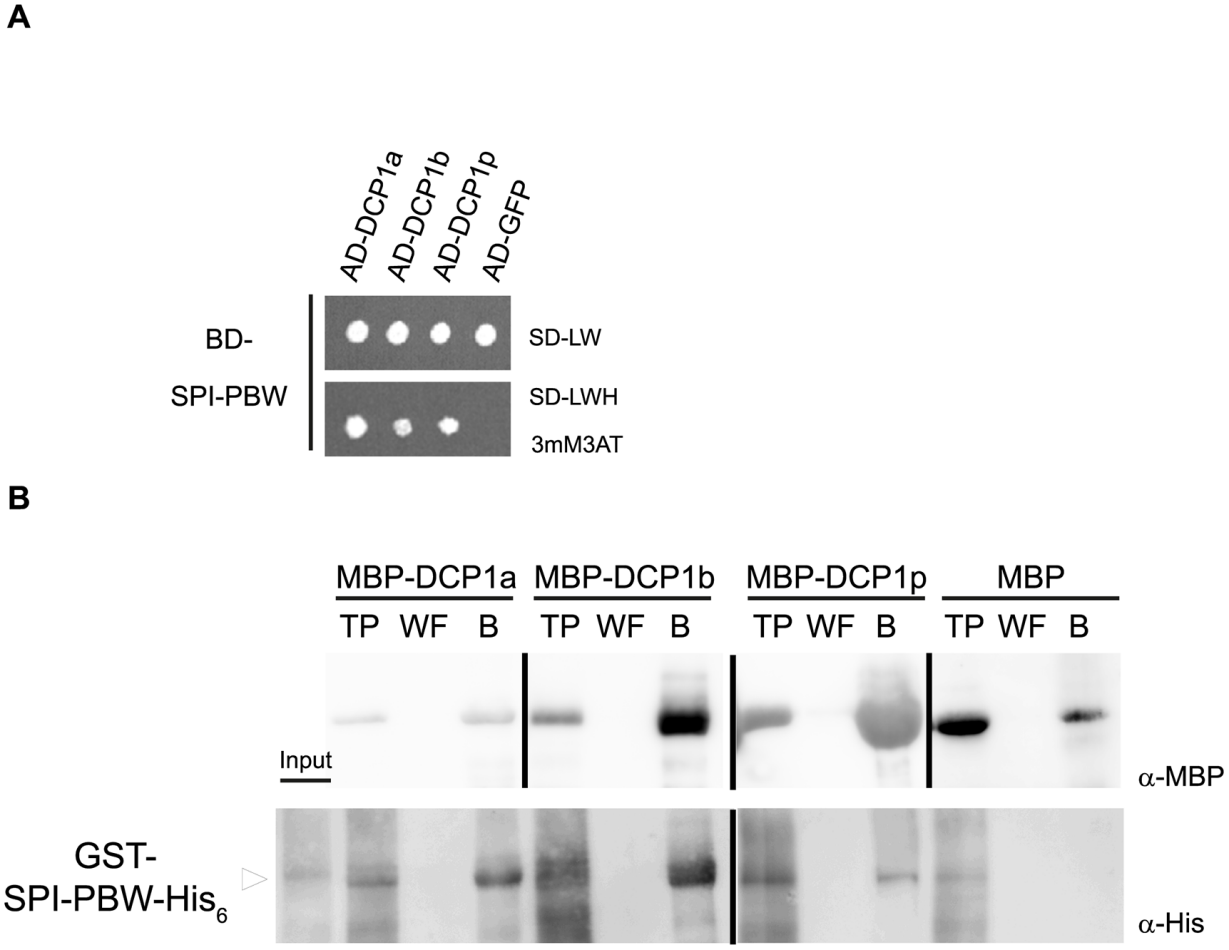
Interspecific interactions between SPI-PBW and DCP1 homologs from mammals and yeast. (A) Yeast two-hybrid interactions. Top part: double transformed yeast cells on selective dropout medium lacking leucine (-L) and tryptophan (-W). Bottom part: interactions between the SPI-PBW N-terminally fused to the GAL4 Binding Domain (BD) and the human DCP1 isoforms (DCP1a and b) and yeast DCP1p, N-terminal fused to the GAL4 Activation Domain (AD) on selective dropout medium lacking leucine (-L), tryptophan (-W) and histidine (-H), supplemented with 3 mM 3-Aminotrizole (3AT). GFP N-terminal fused to the GAL4-AD has been included as negative control. (B) Coprecipitations of bacterially expressed proteins. GST-SPI-PBW-His_6_ (arrowhead, ~110 kDa) coprecipitated with MBP-DCP1a (~90 kDa), MBP-DCP1b (~92kDa) and MBP-DCP1p (~60 kDa), but not with MBP (~42 kDa) as negative control. Throughputs (TP), last wash fractions (WF), and resin bound fractions (B) are visualized by α-MBP (upper row) and α-His_6_ antibody staining (lower row). Samples detected on different blots are separated by lines.

BDCPs and the Decapping machinery are evolutionarily highly conserved and are present in a wide range of eukaryotic organisms. In inter- and intraspecific interaction studies, we also investigated whether an association between BDCPs and P-bodies is evolutionarily conserved. In yeast two-hybrid assays and coprecipitation experiments, we observed the interspecific interactions between the Arabidopsis SPI-PBW and both mammalian DCP1 isoforms (DCP1a and DCP1b) as well as its yeast counterpart (DCP1p) (Fig 10A and 10B). The interaction to the yeast DCP1p was especially surprising, as its protein structure differs strongly from those of mammalian and plant homologs (S12 Fig). In contrast to human and Arabidopsis DCP1 proteins, yeast DCP1p lacks an extended C-terminal domain. In the remaining N-terminal region, we found only one domain, the EVH1 (Enabled/VASP Homology 1 Domain) domain that is conserved in all four DCP1 proteins suggesting that this domain mediates the DCP1 BDCP interactions.

**Fig 11:**
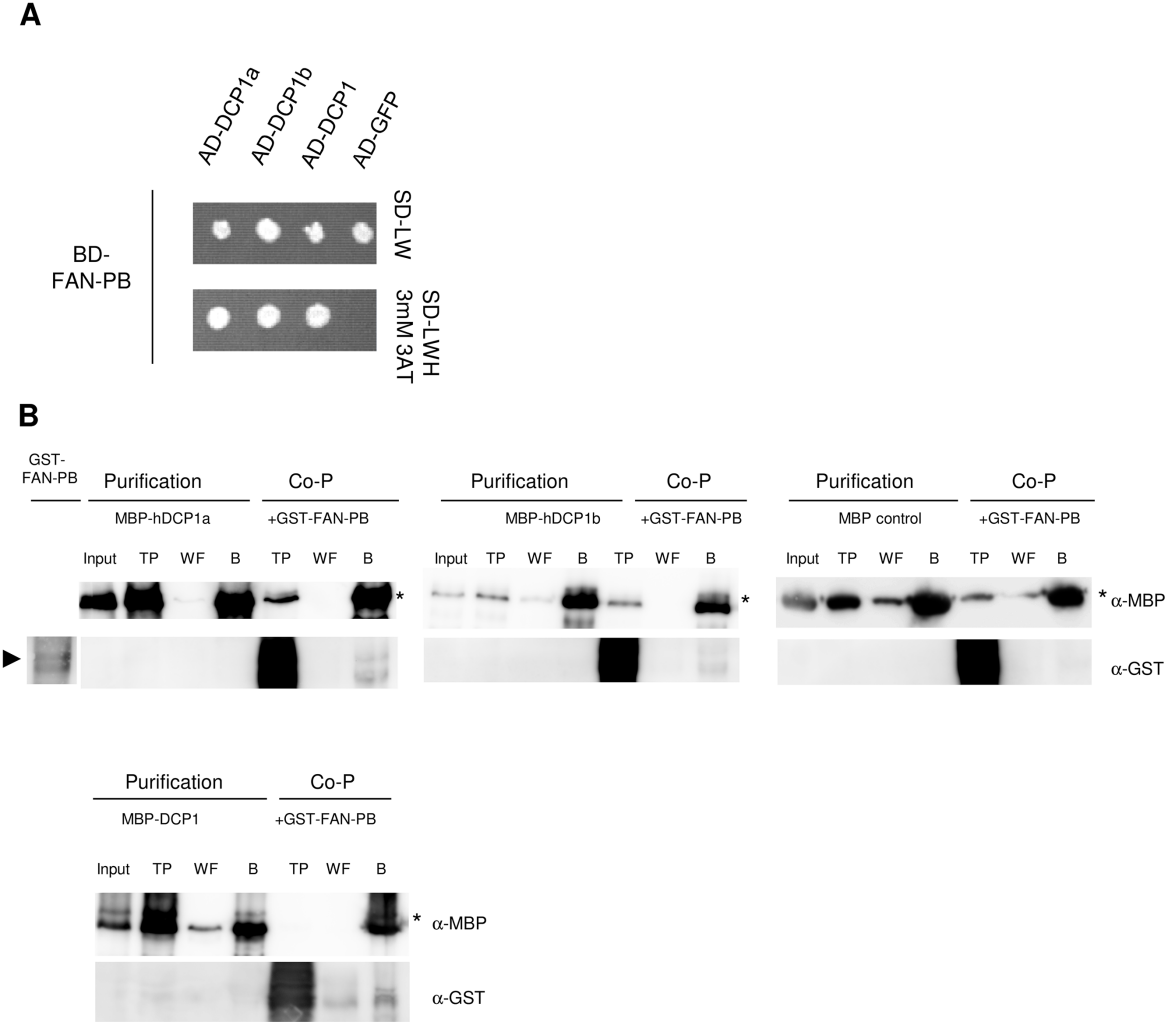
Inter- and intraspecific interactions between FAN-PB and DCP1 homologs. (A) Yeast two-hybrid interactions. Top part: double transformed yeast cells on selective dropout medium lacking leucine (-L) and tryptophan (-W). Bottom part: interactions between the FAN-PB N-terminally fused to the GAL4 Binding Domain (BD), the human DCP1 isoforms (DCP1a and b) and the Arabidopsis DCP1, N-terminally fused to the GAL4 Activation Domain (AD) on selective dropout medium lacking leucine (-L), tryptophan (-W), and histidine (-H), supplemented with 3 mM 3-Aminotrizole (3AT). GFP N-terminal fused to the GAL4-AD has been included as negative control. (B) Coprecipitations of bacterially expressed proteins. GST-FAN-PB (arrowhead, ~70 kDa) coprecipitated with MBP-DCP1a (~90 kDa), MBP-DCP1b (~92 kDa) and MBP-DCP1 from Arabidopsis, but not with MBP (~42 kDa) as negative control. Throughputs (TP), last wash fractions (WF), and resin bound fractions (B) are visualized by α-MBP (upper row) and α-GST antibody staining (lower row). Samples detected on different blots are separated by lines.

To substantiate our hypothesis of an evolutionarily conserved association of BDCPs and the Decapping machinery, we studied the interactions of the PH-BEACH domain containing fragment of the human FAN (Factor Associated with Neutral sphingomyelinase activation) protein with the corresponding human DCP1 isoforms and the Arabidopsis DCP1 homolog. Interactions were found in yeast two-hybrid assays and coprecipitation experiments (Fig 11A and 11B). As we used a C-terminal truncation of FAN lacking its WD40 repeats, we exclude that this protein region is required for an interaction to DCP1. Furthermore, sequence alignments revealed that the eponymous BEACH domain exhibits the highest level of sequence conservation in the C-terminal fragments of SPI and FAN (S13 Fig).

All together our data on intra- and interspecific interactions between Arabidopsis SPI, human FAN, and the DCP1 homologs from Arabidopsis, human, and yeast demonstrate that the association of BDCPs and P-bodies is not plant specific but rather evolutionarily conserved.

## Discussion

In response to various stress conditions, eukaryotic cells can rapidly adapt by reducing their energy consumption, the repression of main protein synthesis [40,62–68], and the regulation of transcript amounts [69–72]. The latter step is achieved by transcriptional as well as post-transcriptional regulatory mechanisms. In this study, we identified the BDCP SPI as a regulator of stress-dependent mRNA stabilization and RNP particle formation. This is unexpected, as to date, BDCPs have been described as facilitators of membrane dynamics and protein sorting in diverse species [1,73–76]. The unique structural interface created by their PBW domain module has been shown to be crucial for their membrane specificities [9]. Accordingly, the plant BDCP SPI is thought to maintain membrane integrity, as *spi* mutants exhibit split vacuoles [2]. We therefore postulate a dual function for the BDCP SPI on membrane dynamics and post-transcriptional regulations.

### Role of SPI in Salt Stress-Dependent Transcriptional Regulation

Col-0 and *spi* react to salt stress with transcript abundance changes related to specific and general stress responses. In comparison to Col-0, *spi* mutants display changes in pathways, including carbohydrate- and polysaccharide-dependent biosynthetic processes, transportations of and response to anorganic and organic substances, immune responses and photosynthesis, pointing to a pleiotropic function of SPI (S3B and S4 Figs). However, we observed similar transcript abundance changes in pathways regulating the responses to wounding, hormones, pathogens and chitin, callose deposition, circadian regulation, metabolic processes, and polysaccharide localization, as well as salinity and water deprivation (Fig 3 and S5–S8 Figs). The comparison of transcriptional changes in a selection of functionally and/or genetically characterized regulators of Arabidopsis salt stress response revealed that most are not affected in *spi* mutants and that few show a lower expression (e.g. ABI1) or a reduced salt stress response (TZF3) (S4 Table). Thus, SPI regulates salt stress-dependent transcriptional regulation in a differential manner.

### SPI Functions as Regulator of Post-transcriptionally Formed RNP Particles and Transcript Localization

The salt stress-dependent relocalization of SPI to P-bodies and its requirement for their assembly suggest a functional role of SPI in post-transcriptional mRNA regulation.

The assembly of microscopically visible RNP particles depends on three processes. First, the amount of cytoplasmic-available mRNA is tightly regulated. Here, translational repression is a prerequisite for all the following steps. This is exemplified by yeast Dhh1p and Pat1p that stimulate P-body formation by translational inhibition [77]. Second, the assembly of RNP particles takes place. This is best shown for the yeast Edc3p and Lsm4p [78,79]. Edc3p and Lsm4p are important scaffold proteins for the assembly of RNP particles and the corresponding mutants display reduced amount of P-bodies. Third, the activity of the mRNA Decapping machinery controls P-body number. Mutations in the decapping enzyme DCP2 or decapping enhancers lead to hyperassembly and enhanced formation of P-bodies [45,80–90]. At what step does SPI contribute to the regulation of P-body formation?

It is unlikely that SPI contributes to P-body formation at the level of translational regulation, as P-body disassembly is similar in wild-type plants and *spi* mutants after CHX treatment (Fig 4A and 4B). In contrast, a role for SPI in the second step, the assembly of P-bodies, is supported by several observations: (1) SPI is recruited to P-bodies in response to salt treatments (Fig 3A and 3B; S1G Fig); (2) P-body formation was greatly impaired after salt treatments in *spi* mutants but increased in wild-type plants (Table 1); (3) the absence of relocalization of TZF3 and RD29B mRNAs to P-bodies under salt stress conditions in *spi* mutants suggests that SPI is important for their recruitment into P-bodies (Table 3). Furthermore, these observations shed new light on the function of P-bodies, which have been mainly described as cytoplasmic spots responsible for transcript decay so far. However, our data suggest that mRNAs can also be stabilized in P-bodies and thereby support recent studies performed in yeast [91,92]; and (4) in contrast to wild-type plants, the number of RD29B mRNA positive granules was not increased upon salt treatments in *spi* mutants, indicating that SPI is required for the assembly of RD29B mRNA containing RNP particles (Table 4).

### BDCPs—General Regulators of RNP Particle Formation?

In interspecific interaction assays, we observed Arabidopsis SPI to physically interact with the core P-body component DCP1 from human and yeast via its structurally conserved PBW module (Figs 1, 10A and 10B; S12 Fig). Therefore, the functions of BDCPs in RNP particle formation and post-transcriptional gene regulation seem to be evolutionarily conserved. This conclusion is supported by our finding that the PH-BEACH domain of human FAN interacts with both isoforms of human DCP1 as well as with the Arabidopsis homolog (Fig 11A and 11B). This conclusion creates a new perspective in understanding the molecular reasons for altered gene expression patterns observed in BDCP mutant mice. For heterozygous NBEA (Neurobeachin) mutant mice, the misregulation of specific hypothalamic genes was reported in response to calorie deprivation [93]. In TNF (Tumor Necrosis Factor)-stimulated FAN-deficient mice, expression of inflammatory genes was selectively impaired [94]. However, the molecular mechanisms causing altered gene abundance remain unclear. An evolutionarily conserved function of BDCPs in stress-dependent RNP particle formation presents an elegant explanation for these findings that are difficult to explain with the current concept of BDCP functioning only in membrane trafficking.

## Materials and Methods

### Plant Material, Growth Conditions, and Stress Treatments

*Arabidopsis thaliana spi-2* (GK_205G08), *spi-3* (SALK_065311), and *spi-4* (GK_420D09) mutants (Columbia ecotype) were obtained from the National Arabidopsis Stock Center. Positions of all T-DNA insertions (S2A Fig) were confirmed by genotyping and sequencing the flanking genomic regions. Loss of expression of full-length transcripts in the T-DNA insertion mutants was confirmed by qualitative RT-PCR spanning the insertion site (S2B Fig). 35S:DCP1-YFP was stably transformed in Col-0, using the *Agrobacterium tumefaciens* strain GV3101 pMP90RK as described previously [95]. Homozygous DCP1-YFP lines in *spi-2* and *spi-4* backgrounds were obtained by crosses. Seeds were surface-sterilized and grown on ½MS medium or on soil under long day conditions at 22°C and 110 μmol m^-2^ s^-1^ light intensity. For salt treatments under transpiring conditions, 18-day-old seedlings (grown on ½MS medium supplemented with 1% sucrose) were transferred to pots containing a sand–soil mixture (9:1; v/v). For the first week plants were watered with ½MS medium only. 24-d-old plants were irrigated daily with ½MS medium (as a control) or ½MS supplemented with NaCl on every second day. The initial NaCl concentration of 50 mM NaCl was either kept or stepped up to 100 mM NaCl after four days of watering, as indicated. Diameter of rosette leaves were taken as indicators for salt tolerance [96] and measured using ImageJ. For root growth inhibition assays under nontranspiring conditions, seeds were transferred to ½MS medium supplemented with different concentrations of NaCl and grown vertically orientated [97]. Root growth rates of salt-treated seedlings were measured 10 d post-transfer and normalized with those of untreated plants. For salt treatments of single transfected cells, rosette leafs of 14- to 16-d-old plants were transferred to culture medium containing different NaCl concentrations for the indicated durations. For blocking translation elongation, rosette leaves were incubated in culture medium containing 0.5 mM CHX (100 mM Stock in 100% DMSO) for the indicated durations [13]. For transcript measurements, 8-d-old vertically grown seedlings were transferred from solid to liquid ½MS (nonstress) medium as well as liquid ½MS medium supplemented with 200 mM NaCl for 4 h. Afterwards, Actinomycin D (ActD) was added to a final concentration of 200 μmol [54]. Plants were kept in the light during incubation. Samples were taken prior to (time point 0) as well as 3 h and 6 h after transcriptional block.

### Plasmids

Coding sequences of *SPI-PBW* (AT1G03060), *DCP1* (AT1G08370), *DCP2* (AT5G13570), DCP5 (AT1G26110), and VCS (AT3G13300) were amplified from Col-0 cDNA; full-length genomic TZF3 (gTZF3;AT4G29190), RD29B (gRD29B; AT5G52300) and ABF3 (gABF3; AT4G34000) were amplified from Col-0 genomic DNA; full-length genomic SPI (gSPI) was cloned by homologous recombination in *E*. *coli* SW102 [98]. In an overlapping PCR, 0.6 kbp of the 5´ and 3´ end of *SPI* ORF were amplified from genomic Arabidopsis Col-0 DNA-adding attB sites and cloned into pDONR207. The resulting plasmid was linearised with *PdmI* and *XbaI* and transformed in *E*. *coli* SW102 containing BAC F10O3 that harbors the complete SPI genomic region. Homologous recombination was induced and gentamycin-positive colonies were analyzed in detail. A clone with the expected restriction pattern was verified by sequencing and used in this study. DCP1p (Q12517), DCP1a (Q9NPI6), and DCP1b (Q8IZD4) were described previously [31]. The PH-BEACH domain-comprising fragment of human FAN (FAN-PB) was amplified from pEGFP-C3:FAN-ΔWD [99]. Primer sequences are provided in S5 Table. Expression vectors containing AtMYC1 and GL1, VPS20.2 and VPS25, were published previously [33,34]. All constructs used in this study were confirmed by sequencing. The following GATEWAY vectors were used for protein expression driven by the CaMV 35S promoter in planta: pENSG or pEXSG-YFP [100] pAMARENA or pAUBERGINE for N- and C-terminal fusions with mCHERRY (M. Jakoby, GenBank ID: FR695418), pCL112 or pCL113 (donated by J. F. Uhrig, S14 Fig, S2 and S3 Texts) for N-terminal fusion with YFP_N_ or YFP_C_, pSCJ232 for N-terminal fusions of 16 BoxB repeats [56]; for protein expression in yeast: pAS or pACT (Clontech); for protein expression in *E*. *coli*: pGEX-2TM-GW (kindly received from Imre Sommsich and Bekir Ülker) for creating fusion proteins with an N-terminal GST- and a C-terminal His_6_-tag, pDEST17 (Invitrogen), pETG-40A (EMBL, Heidelberg, Germany).

### Protein–Protein Interaction Assays

Yeast two-hybrid assays were done as described previously [101]. Interactions were analyzed by selection on synthetic dropout interaction media lacking leucine, tryptophan and histidine, supplemented with 3-Amino-1,2,3-Triazole (3AT). For coprecipitation studies, MBP tagged fusion proteins and MBP as negative control, as well as GST or GST/His_6_-tagged proteins were expressed in *E*. *coli* (BL21 (DE3) RIL). Bacteria were grown in TB medium (37°C, OD_600_ = 1), induced by isopropyl b-D-1-thiogalactopyranosie (IPTG) and incubated (20°C, 6 h). Cells were harvested and resuspended in TRIS purification buffer (100 mM TRIS, 150 mM NaCl, 1% Triton X-100, pH 8.0). After addition of Lysozyme (100 μg/ml) and incubation on ice (20 min), samples were sonicated. Lysates of MBP fusion proteins were cleared by centrifugation (4,000 g, 15 min, 4°C). Amylose resin was labeled according to the manufactures’ instructions (New England Biolabs). Prior to centrifugation of lysates of SPI-PBW- and FAN-PB-fusion proteins (10,000 g, 5 min, 4°C), N-Lauroylsarcosine sodium salt (Sigma-Aldrich) was added (1% final concentration). Triton X-100 was added to the cleared lysates of SPI-PBW- and FAN-PB-fusion proteins (1% final concentration). Labeled amylose resins were incubated in cleared lysates of SPI-PBW-or FAN-PB-fusion proteins (1 h, 4°C) under constant shaking. MBP-fusion proteins were purified according to the manufacturer’s instructions (New England Biolabs) using TRIS purification buffer for all wash steps. Purifications and coprecipitations were analyzed by immunoblotting as described previously [31].

### Transient Expression in Plants

*Nicotiana benthamiana* leaves were transiently transformed by infiltration with *Agrobacterium tumefaciens* (GV3101 pMP90RK). Coinfiltrated cultures were mixed in equal proportions (1:1:1) and incubated for 4 h at RT [102]. Transfection of Arabidopsis leaves was performed by biolistic transformation [103] and analyzed after 12 to 16 h by Confocal Laser Scanning Microscopy (CLSM).

### CLSM and Image Evaluation

CLSM was done as described previously [31]. Colocalization of dot-like structures was analyzed manually. For BiFC analysis, cells were considered to be transfected when a cotransfected marker protein was expressed. As negative controls, noninteracting proteins that are found in the cytoplasm were included [104]. In three biological replicates (*n* = 30 cells), no YFP reconstruction was observed. To ensure comparability between transfected cells, laser intensities and exposure times were fixed and no automated corrections used.

### FRET-AP Assays

Transfected leaf epidermis cells were analyzed for FRET between 35S_pro_:DCP1-CFP (donor molecule) and 35S_pro_:YFP-gSPI (acceptor molecule) using a Leica SP8 confocal microscope, equipped with a 20 x 0.75 objective (HC PL AP IMM CORR C52) at a 512 x 512 resolution format. Pre and post-AP of the emission spectra of the donor and acceptor were recorded by sequential scanning at 475 nm +/- 10 nm upon excitation at 458 nm and at 540 nm +/- 15 nm upon excitation at 514 nm in z-stacks of whole transfected cells, respectively. The detection of donor and acceptor emissions occurred via high-efficiency hybrid detectors. Laser intensities of 1.3% (DCP1-CFP), 10.7% (for YFP-gSPI), or 0.5% (for free YFP) were fixed. Targeted AP was done at 514 nm with 60% laser intensity of a 30% activated Argon laser on a defined region of interest (ROI) covering the whole cell and parts of the background by scanning through the complete *z*-axis of the selected part in 1 μm steps. Photodestruction of the acceptor was 40% on average (+/- 6%). For each cell, maximum projections of z-stacks pre- and post-AP were created and fluorescence intensities of whole cell areas and stationary PBs were measured separately by setting defined ROIs manually using the quantification tool of the LAS AF (Leica Application Suite Advanced Fluorescence 2.4.1) software. The intensity of an untransfected leaf area (pre- and post-AP) was measured and subtracted from the donor intensities as background corrective. Cells transfected with 35S_pro_:DCP1-CFP only were treated similarly to double transfected cells to determine the photobleaching corrective of the donor molecule in the whole cell (-6.38%; *n* = 14) and in stationary PBs (-8.4%; *n* = 50). Donor emissions after AP were collectively photobleaching corrected (Intensity Donor emission post-AP_(corr)_) [105]. The FRET_E_ was determined according to the calculation:

$$\text{E}\;=\;(\;1-\frac{\left(Intensity\;Donor\;emission\;prior\;to\;AP\;-Background\;Intensity\right)}{\left(Intensity\;Donor\;emission\;post\;AP\;\left(corr\right)-\;Background\;Intensity\right)}\;).$$

### Transcript Analysis and Determination of mRNA Decay

Total RNA was isolated (Qiagen, RNeasy Mini kit), treated with DNaseI (Thermoscientific) according to the manufacturer´s recommendations, and quantified spectrophotometrically. 1 μg of total RNA was reverse-transcribed (SuperScriptIII, Invitrogen). qPCRs were performed on an Applied Biosystems 7,300 real-time PCR system (http://www.appliedbiosystems.com) using POWER SYBR Green PCR-Master Mix (Applied Biosystems). Transcript levels were normalized to the 18S rRNA [106]. All qPCR data represent the average of three biological and two technical replicates. For determination of mRNA decay, Ct values after transcription inhibition were normalized to those of 18S rRNA and compared further to their normalized values prior to Actinomycin D treatment. Primer sequences are provided in S6 Table.

### RNA Sequencing and Transcriptome Analysis

Isolated RNA was checked for RNA integrity (RIN > 7, Agilent 2010 Bioanalyzer [Agilent]) and prepared for Illumina sequencing using the TruSeq RNA sample prep kit versus Illumina. The resulting libraries were sequenced by Illumina HiSeq according to the manufacturers protocol using the Beckmann Coulter service as 12 samples multiplexed on one lane (please find the complete data set under http://www.ncbi.nlm.nih.gov/bioproject/278120).

The resulting reads were mapped against the Arabidopsis genome with the TAIR10 gff files for annotation using CLC genomics workbench (Quiagen), and total reads per gene were extracted as the measure for gene expression (S7 Table). Differential expression was called using the Bioconductor package edgeR [107]. Read counts were normalized to reads per mapped million, averages were calculated, and log-fold changes were calculated adding one value to avoid division by zero. Gene IDs were functionally annotated using the descriptions from TAIR10 (www.arabidopsis.org) and the MapMan annotation (S8 Table) [108]. Functional enrichments were calculated based on GO terms using GOrilla [109] using the significantly regulated genes as the target group and all genes tested as the background. *p*-values are calculated using the hypergeometric distribution and corrected with Benjamini Hochberg for multiple hypotheses testing. Additional functional enrichments were calculated for the metabolism-centric MapMan categories using the embedded Wilcoxon sum rank algorithm with Benjamini Hochberg correction [110]. Using both categorizations for enrichments exploits the strength of the GO annotation (regulation) and the strength of the MapMan categorization (metabolism).

## Supporting Information

S1 Data Excel spreadsheet containing, in separate sheets, the corresponding numerical and statistical data for the panels of Figs 1D, 4B, 5A and 5B, 5D, 7A–7D, 8A–8D and S1E, S1G–S1H, S2C and S2D, S2F, S10A–S10F, and S11A–S11F Figs. (XLSX)

S1 Fig BiFC negative controls and subcellular localization of SPI. (A) Cytoplasmic localization of VPS20.2-mCHERRY and (B) AtMYC1-CFP in transiently transformed *N*. *benthamiana* leafs. Left panel shows the corresponding transmission pictures. (C) BiFC interaction between VPS20.2-YFP_N_ and VPS25-YFP_C_ and (D) YFP_C_-AtMYC1 and YFP_N_-GL1 cotransfected with free RFP as a transformation control. Scale bar: 35 μm. (E) Percentage of cells showing YFP-gSPI in cytoplasmic dot-like structures in single or DCP1-mCHERRY cotransfected cells. Error bars represent standard deviations of three biological replicates (each 20 cells). Two-tailed student’s *t* tests were performed to compare single and double transfected cells (*** *p <* 0.001). Note that the data set for nonstress conditions also serves as a reference in part C and D of this Figure. (F) Recruitment of YFP-gSPI (left) to DCP1-mCHERRY-labeled P-bodies (middle) under nonstress conditions. Right picture shows the overlay. Scale bar: 50 μm. (G) Percentage of cells showing a relocalization of YFP-gSPI to P-bodies at increasing salt concentrations (½MS + indicated NaCl concentration for 10 h). Data denote the average from three independent biological replicates (each 20 cells). Error bars represent standard deviations. Two-tailed student’s *t* tests were performed to compare nonstress and salt stress conditions (** *p* < 0.01; *** *p* < 0.001). (H) Relocalization of YFP-gSPI to P-bodies was quantified under nonstress (½MS) and osmotic stress conditions (½MS + 125 or 300 mM Mannitol (Man) for 10 h). Data denote the average from three independent biological replicates (each at least 20 cells). Error bars represent standard deviations. No significant changes between nonstress and osmotic stress conditions were observed (two-tailed student’s *t* tests). (I) Cytoplasmic distribution of free YFP under nonstress (½MS) and salt stress conditions (½MS + 140mM NaCl for 10 h) (*n* = 30 cells). Scale bar: 50 μm.
(TIF)

S2 Fig *spi* mutants. (A) Schematic presentation of *SPIRRIG* T-DNA insertion lines. (B) T-DNA insertions in *spi-2*, *spi-3*, and *spi-4* were confirmed by qualitative RT-PCR with a primer pair spanning the insertion side (I) and second pair covering the coding region before the insertion up to exon located T-DNA (T). cDNA from Col-0 plants and H_2_O were included as controls. M = 1 kb plus ladder (Invitrogen). (C) Change of P-body number (in %) after mock treatments (½MS supplemented with 0.5% DMSO for 90 min). Data denote the average from three independent biological replicates (each three whole leaf areas). Error bars represent standard deviations. No significant changes between P-body numbers before and after the treatment could be determined (two-tailed student’s *t* tests). (D) Change of P-body number (in %) in leaf epidermis cells transiently transfected with DCP2-mCHERRY after an incubation of 90 min in ½MS supplemented with 0.5 mM CHX. Data denote the average from three independent biological replicates (each three cells). Error bars represent standard deviations. Two-tailed student’s *t* tests were performed to compare cells before and after the treatment (* *p* < 0.05; ** *p* < 0.01). (E) Representative images of YFP-gSPI (left) colocalizing with DCP1-mCHERRY labeled P-bodies (middle) 150 min after continuous CHX treatment. Right picture presents the overlay. Scale bar: 50 μm. (F) Change of P-body number (in %) in leaf epidermis cells transiently transfected with DCP1-YFP after simultaneous treatment with 0.5 mM and 140 mM NaCl for 90 min. Data denote the average from three independent biological replicates (each three cells). Error bars represent standard deviations. Two-tailed student’s *t* tests were performed to compare cells before and after the treatment (* *p* < 0.05).
(PDF)

S3 Fig Comparison of transcriptional response between Col-0 and *spi*. A) Principle component analysis (PCA) presenting Col-0 in blue circles, salt stress-treated Col-0 in blue squares, *spi* in red circles and salt stress-treated *spi* in red squares. The 1st dimension explains 76.4%, the 2nd dimension 6.5%. B) Mapman visualization of log2-fold changes comparing the transcriptional pattern between Col-0 and *spi* upon salt stress induction.
(PDF)

S4 Fig Significantly lower enriched GO-Terms in *spi* mutants under salt stress conditions in comparison to Col-0. Darker colors in GO term categories represent higher q-values (BH-corrected).
(PDF)

S5 Fig GO-Term enrichments of genes up-regulated in Col-0 under salt stress conditions. Darker colors in GO term categories represent higher q-values (BH-corrected).
(PDF)

S6 Fig GO-Term enrichments of genes down-regulated in Col-0 under salt stress conditions. Darker colors in GO term categories represent higher q-values (BH-corrected).
(PDF)

S7 Fig GO-Term enrichments of genes up-regulated in *spi* under salt stress conditions. Darker colors in GO term categories represent higher q-values (BH-corrected).
(PDF)

S8 Fig GO-Term enrichments of genes down-regulated in *spi* under salt stress conditions. Darker colors in GO term categories represent higher q-values (BH-corrected).
(PDF)

S9 Fig Salt stress-regulated transcriptional changes in Col-0 and *spi*. (A) Box Whisker Plot of the fold-change for 3,400 up-regulated genes (left) and 2,611 down-regulated genes (right) in salt-treated Col-0 and *spi* mutants. (B) Venn diagram comparing the salt stress-dependent down-regulation of transcripts in Col-0 and *spi*.
(PDF)

S10 Fig mRNA stability and granule formation in *spi* mutants. (A) Distribution of LambdaN22-mVENUS reporter coexpressed with the BoxB without any mRNA target under nonstress (½MS) and salt stress (NaCl; ½MS supplemented with 140 mM NaCl for 10 h) conditions in transfected leaf epidermis cells. Data denote the average of 60 cells. Error bars represent standard deviations. Distribution of LambdaN22-mVENUS reporter coexpressed with (B) RD29B mRNA N-terminally fused to mCHERRY (*n* = 30 cells) and (C) TZF3 mRNA N-terminally fused to mCHERRY (*n* = 120 cells) under nonstress (½MS) conditions. Error bars represent standard deviations. (D) mRNA stabilities of ABF3 (in %) were determined 3 h and 6 h after application of Actinomycin D (ActD) relative to time point 0 under nonstress (½MS) and salt stress conditions (200 mM NaCl in ½MS liquid medium for 4 h). Data denote the average from three independent biological and two technical replicates. Error bars represent the standard error of the mean. No significant changes between wild-type and *spi* mutants could be determined (two-tailed student’s *t* tests). (E) Distribution of LambdaN22-mVENUS reporter coexpressed with gABF3 N-terminally fused to BoxB repeats and with (F) gABF3 N-terminally fused to mCHERRY under nonstress (½MS) and salt stress (NaCl) conditions.
(PDF)

S11 Fig mRNAs of TZF3 and RD29B accumulate in cytoplasmic granules. Data denote the average number of cells (in %) showing cytoplasmic accumulations of the LamdaN22-mVENUS reporter in cells coexpressing (A) 16BoxB-gRD29B (*n* = 65 cells), (B) 16BoxB-gTZF3 (*n* = 150 cells) under nonstress (-NaCl) and salt stress (NaCl; ½MS supplemented with 140 mM NaCl for 10 h) conditions. Presented are the averages from four biological replicates of RD29B (*n* = 20 cells each) and seven biological replicates of TZF3 (*n* = 20 cells each). (C) Distribution of LambdaN22-mVENUS reporter before and after simultaneous treatments with 0.5mM CHX and 140 mM NaCl for 90 min in cells cotransfected with 16BoxB-gRD29B (*n* = 10 cells per replicate) and (D) 16B-gTZF3 (*n* = 15 cells per replicate). Presented are the averages from three biological replicates. Error bars represent standard deviations. Two-tailed student’s *t* tests were performed to compare nonstress and salt stress conditions (* *p* < 0.05).
(PDF)

S12 Fig Comparative alignment of sequences from Arabidopsis (AtDCP1), human (HsDCP1a, HsDCP1b), and yeast (ScDCP1p) DCP1 homologs. Residues forming the aromatic triade in the hydrophobic cleft in the EVH1 domain (underlined with a dark grey bar) are in bold. Asterisks highlight positions that have a single, fully conserved residue. Colons indicate conservation between groups of strongly similar properties—scoring >0.5 in the Gonnet PAM 250 matrix. Periods represent conservation between groups of weakly similar properties—scoring <0.5 in the Gonnet PAM 250 matrix.
(PDF)

S13 Fig Alignment of the C-terminal sequences of SPIRRIG and FAN. The GRAM and PH domains of FAN and SPI are shaded in dark and light gray, respectively. The BEACH domain in both proteins is underlined. The region containing WD40 repeats is printed in bold; the part that is not included in the clone used in this study is in italic letters. Asterisks highlight positions that have a single, fully conserved residue. Colons indicate conservation between groups of strongly similar properties—scoring >0.5 in the Gonnet PAM 250 matrix. Periods represent conservation between groups of weakly similar properties—scoring <0.5 in the Gonnet PAM 250 matrix.
(PDF)

S14 Fig Maps of BiFC vectors for N-terminal fusions of YFP-fragments. Annotated drawing of Gateway®- compatible A) pCL112- and B) pCL113- vectors used for BiFC assays (donated by Joachim Uhrig).
(PDF)

S1 Table Quantification of BiFC assays in cells coexpressing DCP2-mCHERRY. Total numbers of analyzed cells are provided.
(DOCX)

S2 Table Quantification of BiFC assays in cells coexpressing free RFP. Total numbers of analyzed cells are provided.
(DOCX)

S3 Table Comparison of hyperosmotic salinity and salt stress response GO term categories. Differentially enriched transcripts in Col-0 and *spi* are highlighted by black boxes.
(DOCX)

S4 Table Gene expression pattern of selected salt stress-regulated transcripts. Displayed are the mean expression values and for 19 salt stress-responsive genes in Col-0 (wt) and *spi* (mut) mutants under nonstress and salt stress (NaCl) conditions. Expression changes between control and salt stress (NaCl) conditions as well as Col-0 and *spi* are presented as log-fold change. Statistically significant changes between *spi* and Col-0 are highlighted by red boxes (q-values, BH-corrected).
(DOCX)

S5 Table Sequences of primers used for cloning. (DOCX)

S6 Table Sequences of primers used for qPCR analysis. (DOCX)

S7 Table Additional information on RNA-Seq analysis. Presented are the numbers of total fragments, mapped fragments, uniquely mapped fragments, nonspecific mappings, unmapped fragments, mapped exons, spanned exon–exon borders, total exons, annotated introns, and the number of sequenced genes per sample.
(DOCX)

S8 Table Whole data set describing gene expression levels in Col-0 and *spi* under nonstress and salt stress conditions (please see separate excel file). Additional sheets present up-regulated in Col-0 under salt stress conditions; transcripts down-regulated in Col-0 under salt stress conditions; transcripts up-regulated in *spi* under salt stress conditions; transcripts down-regulated in *spi* under salt stress conditions; transcripts down-regulated in nontreated *spi* in comparison to nontreated Col-0; transcripts up-regulated in nontreated *spi* in comparison to nontreated Col-0; transcripts down-regulated in salt-treated *spi* in comparison to salt-treated Col-0; transcripts up-regulated in salt-treated *spi* in comparison to salt-treated Col-0.
(XLSX)

S1 Text Additional information about RNA-Seq analysis. General transcriptional changes between Col-0 and *spi* under nonstress conditions and salt stress-dependent transcriptional changes in Col-0 and *spi* are presented.
(DOCX)

S2 Text Fasta sequence of Gateway-compatible pCL112-vector. (TXT)

S3 Text Fasta sequence of Gateway-compatible pCL113-vector. (TXT)

## Abbreviations:

AP: acceptor photobleaching
BDCP: BEACH domain containing proteins
BEACH: *beige* and Chediak Higashi
BiFC: Bimolecular Fluorescence Complementation
CHS: Chediak Higashi Syndrome, CHX, Cycloheximide
FRET: Förster-Resonance Energy Transfer
GO: Gene Ontology
MS: Murashige and Skoog
P-body: mRNA processing body
PBW: PH-BEACH-WD40
PCA: principle component analysis
PH: Pleckstrin-Homology
RNP: ribonucleoprotein
