# Supplementary figures and images for: The BEACH Domain Protein SPIRRIG Is Essential for Arabidopsis Salt Stress Tolerance and Functions as a Regulator of Transcript Stabilization and Localization
Source: PLoS Biol. 2015 Jul 2;13(7):e1002188. doi: 10.1371/journal.pbio.1002188 (PMC4489804; doi:10.1371/journal.pbio.1002188)

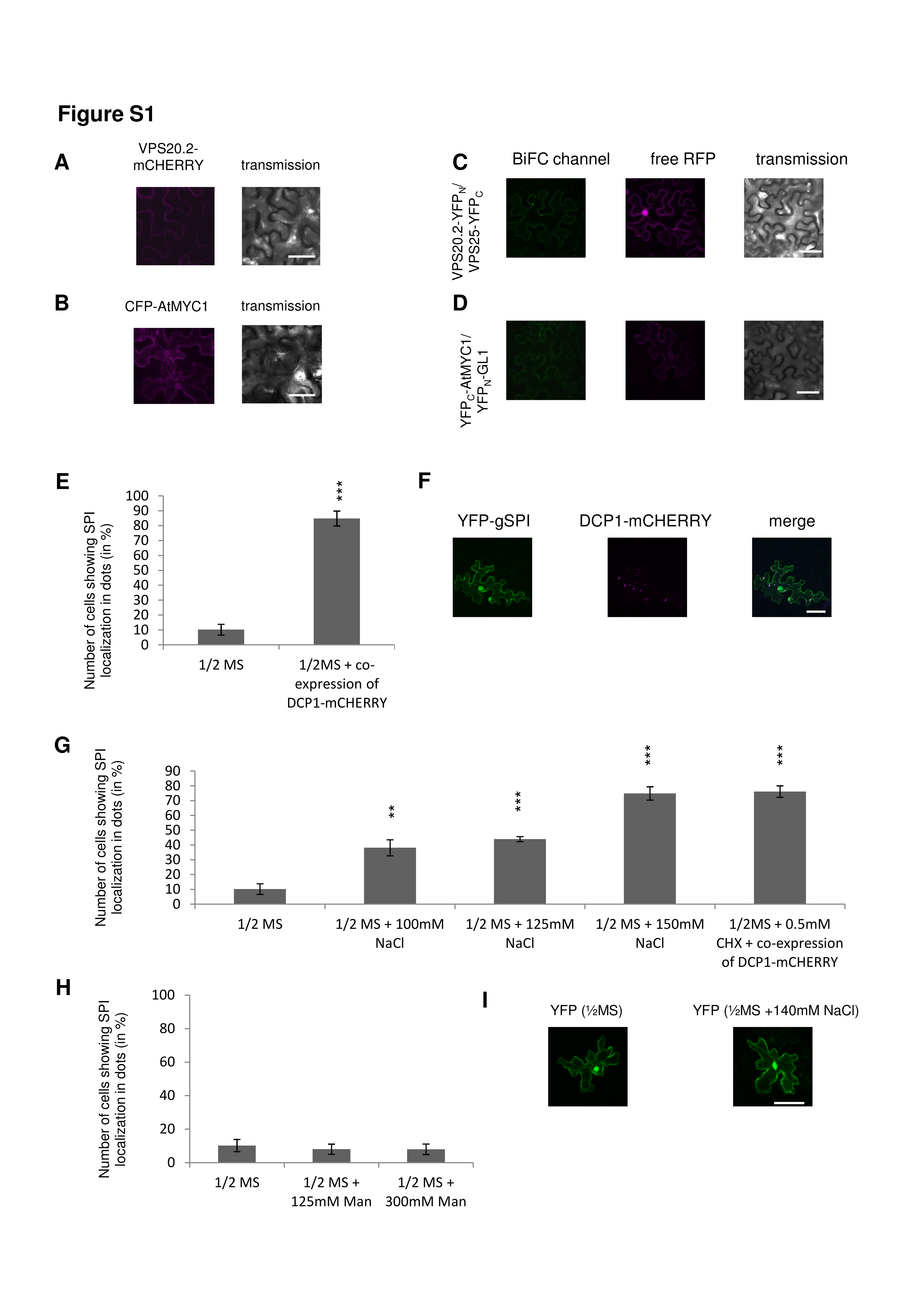

Supplement: S1 Fig — (A) Cytoplasmic localization of VPS20.2-mCHERRY and (B) AtMYC1-CFP in transiently transformed N. benthamiana leafs. Left panel shows the corresponding transmission pictures. (C) BiFC interaction between VPS20.2-YFPN and VPS25-YFPC and (D) YFPC-AtMYC1 and YFPN-GL1 cotransfected with free RFP as a transformation control. Scale bar: 35 μm. (E) Percentage of cells showing YFP-gSPI in cytoplasmic dot-like structures in single or DCP1-mCHERRY cotransfected cells. Error bars represent standard deviations of three biological replicates (each 20 cells). Two-tailed student’s t tests were performed to compare single and double transfected cells (*** p < 0.001). Note that the data set for nonstress conditions also serves as a reference in part C and D of this Figure. (F) Recruitment of YFP-gSPI (left) to DCP1-mCHERRY-labeled P-bodies (middle) under nonstress conditions. Right picture shows the overlay. Scale bar: 50 μm. (G) Percentage of cells showing a relocalization of YFP-gSPI to P-bodies at increasing salt concentrations (½MS + indicated NaCl concentration for 10 h). Data denote the average from three independent biological replicates (each 20 cells). Error bars represent standard deviations. Two-tailed student’s t tests were performed to compare nonstress and salt stress conditions (** p < 0.01; *** p < 0.001). (H) Relocalization of YFP-gSPI to P-bodies was quantified under nonstress (½MS) and osmotic stress conditions (½MS + 125 or 300 mM Mannitol (Man) for 10 h). Data denote the average from three independent biological replicates (each at least 20 cells). Error bars represent standard deviations. No significant changes between nonstress and osmotic stress conditions were observed (two-tailed student’s t tests). (I) Cytoplasmic distribution of free YFP under nonstress (½MS) and salt stress conditions (½MS + 140mM NaCl for 10 h) (n = 30 cells). Scale bar: 50 μm. (TIF) [file pbio.1002188.s002.tif]

**Figure S2**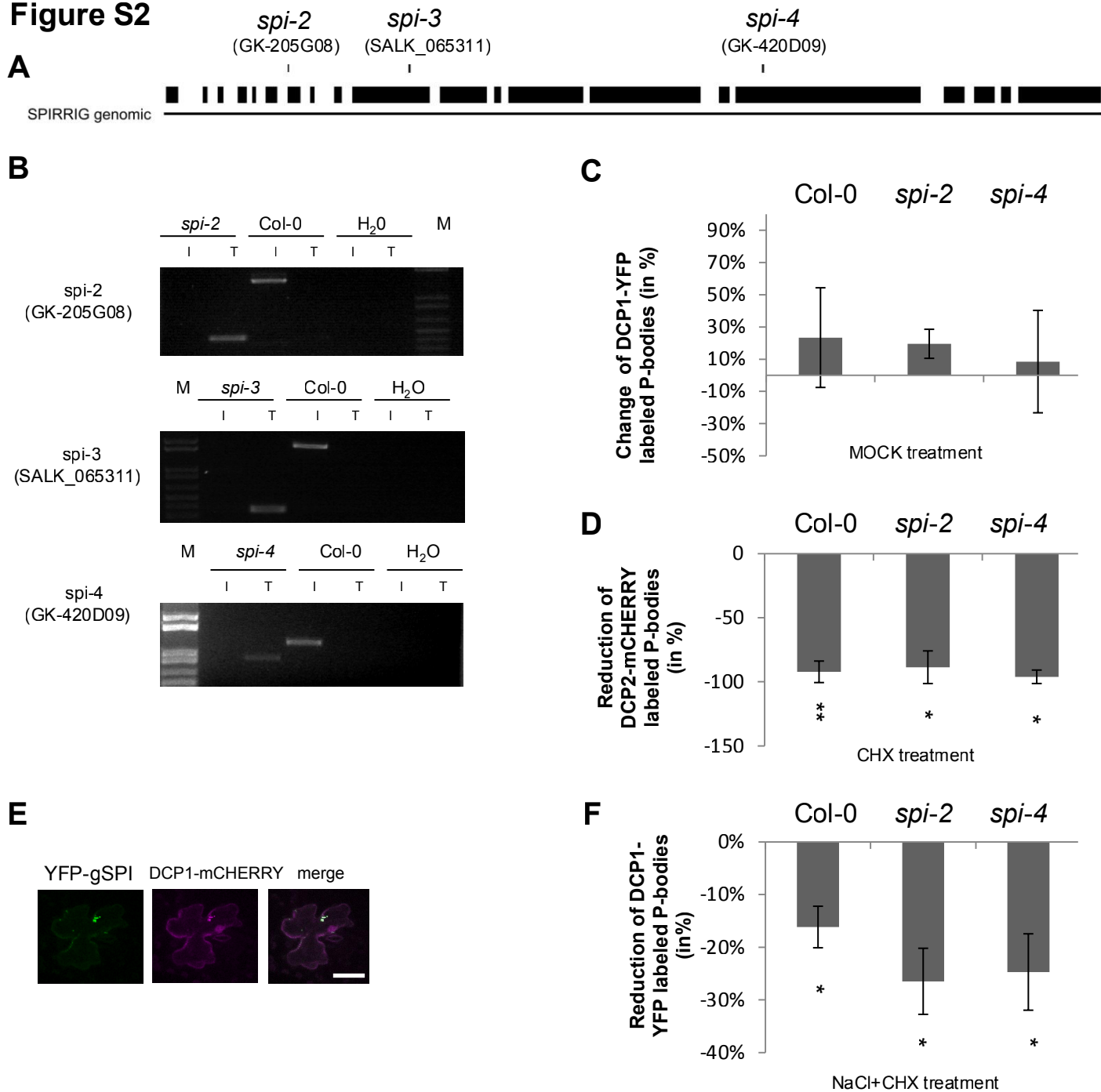

Supplement: S2 Fig — (A) Schematic presentation of SPIRRIG T-DNA insertion lines. (B) T-DNA insertions in spi-2, spi-3, and spi-4 were confirmed by qualitative RT-PCR with a primer pair spanning the insertion side (I) and second pair covering the coding region before the insertion up to exon located T-DNA (T). cDNA from Col-0 plants and H2O were included as controls. M = 1 kb plus ladder (Invitrogen). (C) Change of P-body number (in %) after mock treatments (½MS supplemented with 0.5% DMSO for 90 min). Data denote the average from three independent biological replicates (each three whole leaf areas). Error bars represent standard deviations. No significant changes between P-body numbers before and after the treatment could be determined (two-tailed student’s t tests). (D) Change of P-body number (in %) in leaf epidermis cells transiently transfected with DCP2-mCHERRY after an incubation of 90 min in ½MS supplemented with 0.5 mM CHX. Data denote the average from three independent biological replicates (each three cells). Error bars represent standard deviations. Two-tailed student’s t tests were performed to compare cells before and after the treatment (* p < 0.05; ** p < 0.01). (E) Representative images of YFP-gSPI (left) colocalizing with DCP1-mCHERRY labeled P-bodies (middle) 150 min after continuous CHX treatment. Right picture presents the overlay. Scale bar: 50 μm. (F) Change of P-body number (in %) in leaf epidermis cells transiently transfected with DCP1-YFP after simultaneous treatment with 0.5 mM and 140 mM NaCl for 90 min. Data denote the average from three independent biological replicates (each three cells). Error bars represent standard deviations. Two-tailed student’s t tests were performed to compare cells before and after the treatment (* p < 0.05). (PDF) [file pbio.1002188.s003.pdf]

Figure S3

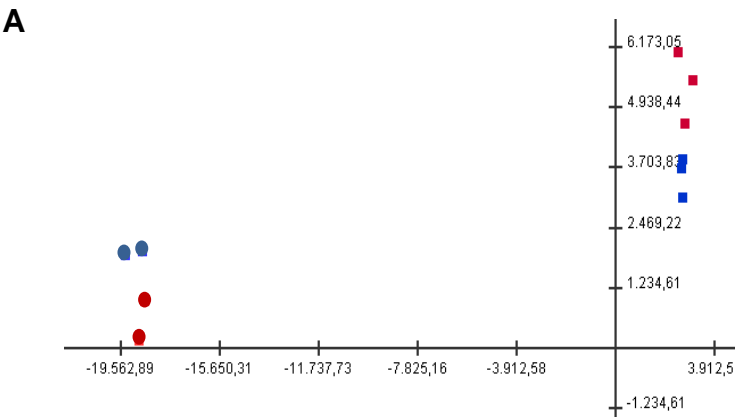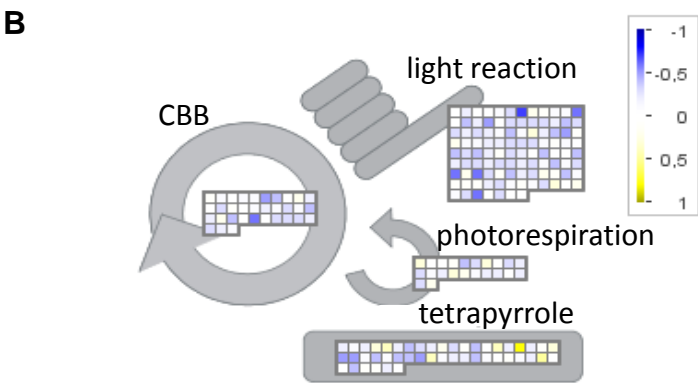

Supplement: S3 Fig — A) Principle component analysis (PCA) presenting Col-0 in blue circles, salt stress-treated Col-0 in blue squares, spi in red circles and salt stress-treated spi in red squares. The 1st dimension explains 76.4%, the 2nd dimension 6.5%. B) Mapman visualization of log2-fold changes comparing the transcriptional pattern between Col-0 and spi upon salt stress induction. (PDF) [file pbio.1002188.s004.pdf]

### Figure S4

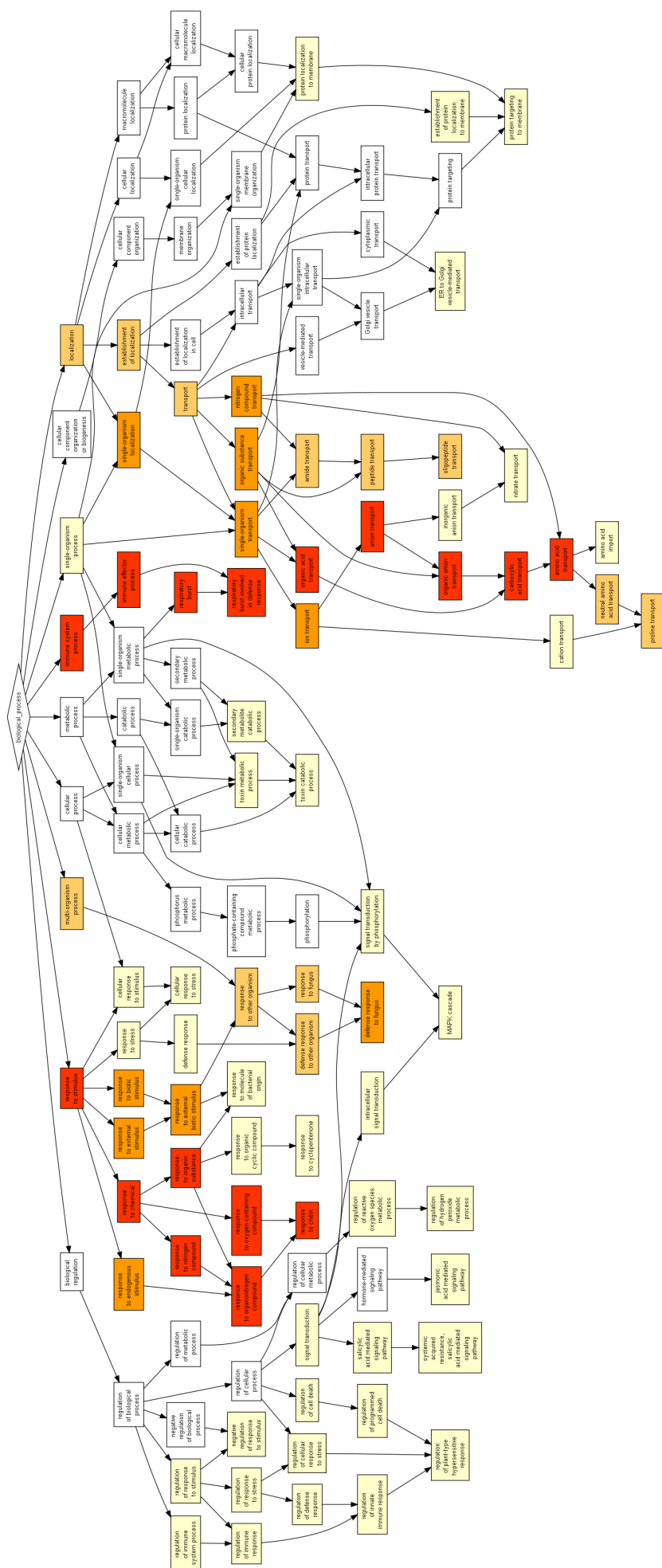

Supplement: S4 Fig — Darker colors in GO term categories represent higher q-values (BH-corrected). (PDF) [file pbio.1002188.s005.pdf]

Figure S5

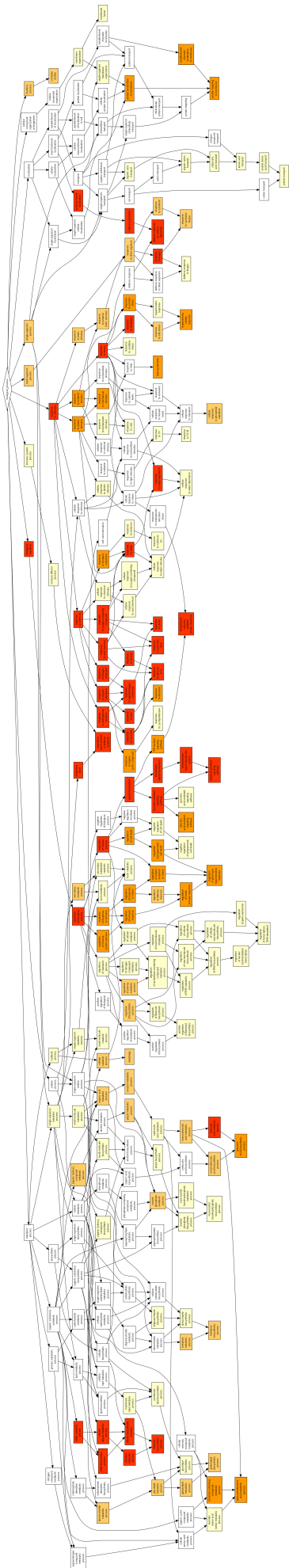

Supplement: S5 Fig — Darker colors in GO term categories represent higher q-values (BH-corrected). (PDF) [file pbio.1002188.s006.pdf]

Figure S6

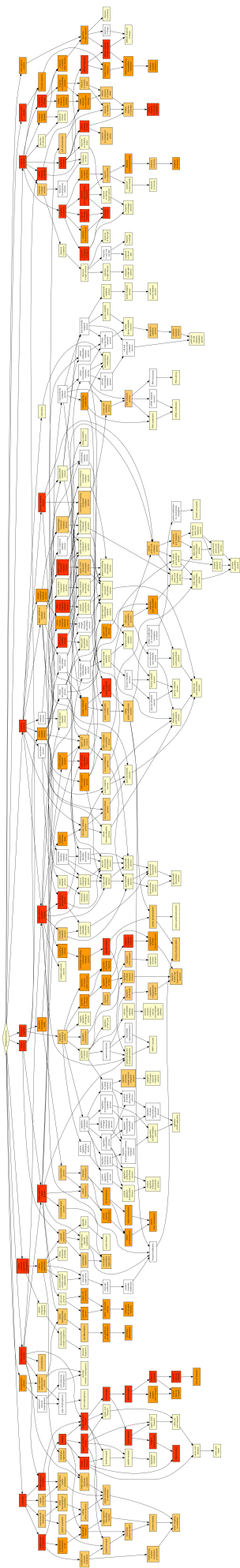

Supplement: S6 Fig — Darker colors in GO term categories represent higher q-values (BH-corrected). (PDF) [file pbio.1002188.s007.pdf]

Figure S7

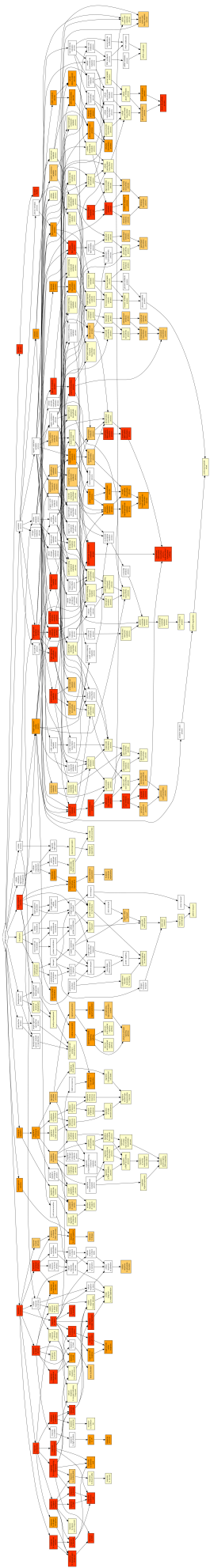

Supplement: S7 Fig — Darker colors in GO term categories represent higher q-values (BH-corrected). (PDF) [file pbio.1002188.s008.pdf]

Figure S8

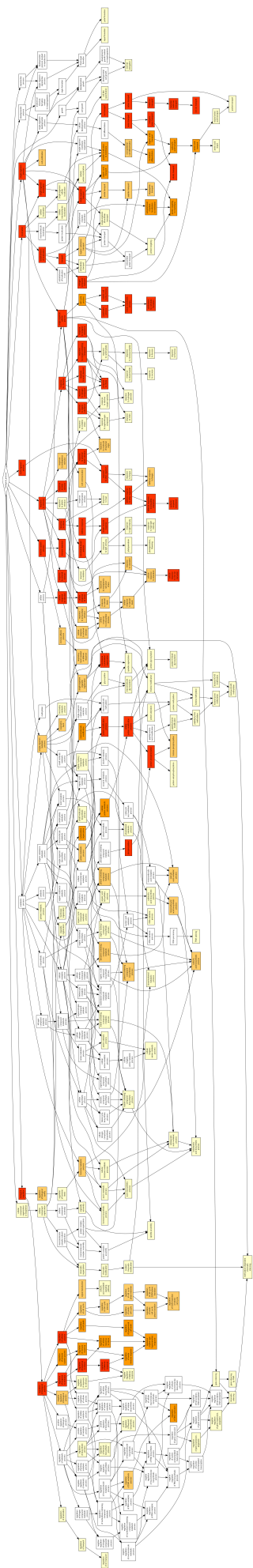

Supplement: S8 Fig — Darker colors in GO term categories represent higher q-values (BH-corrected). (PDF) [file pbio.1002188.s009.pdf]

Figure S9

A)

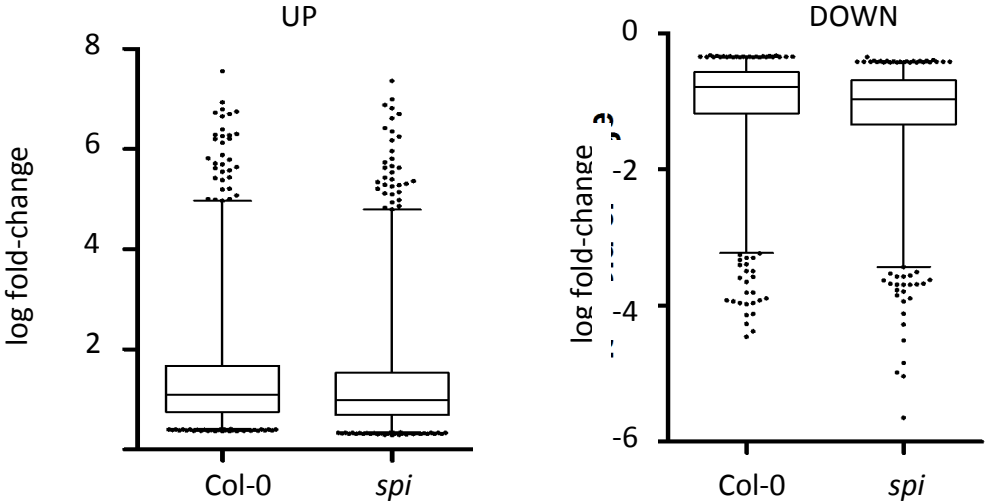

B)

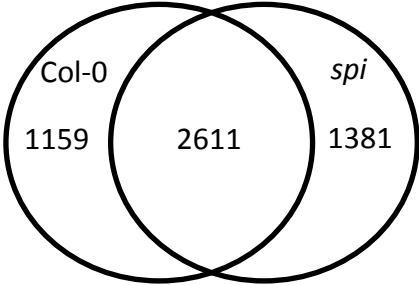

Supplement: S9 Fig — (A) Box Whisker Plot of the fold-change for 3,400 up-regulated genes (left) and 2,611 down-regulated genes (right) in salt-treated Col-0 and spi mutants. (B) Venn diagram comparing the salt stress-dependent down-regulation of transcripts in Col-0 and spi. (PDF) [file pbio.1002188.s010.pdf]

**Figure S10**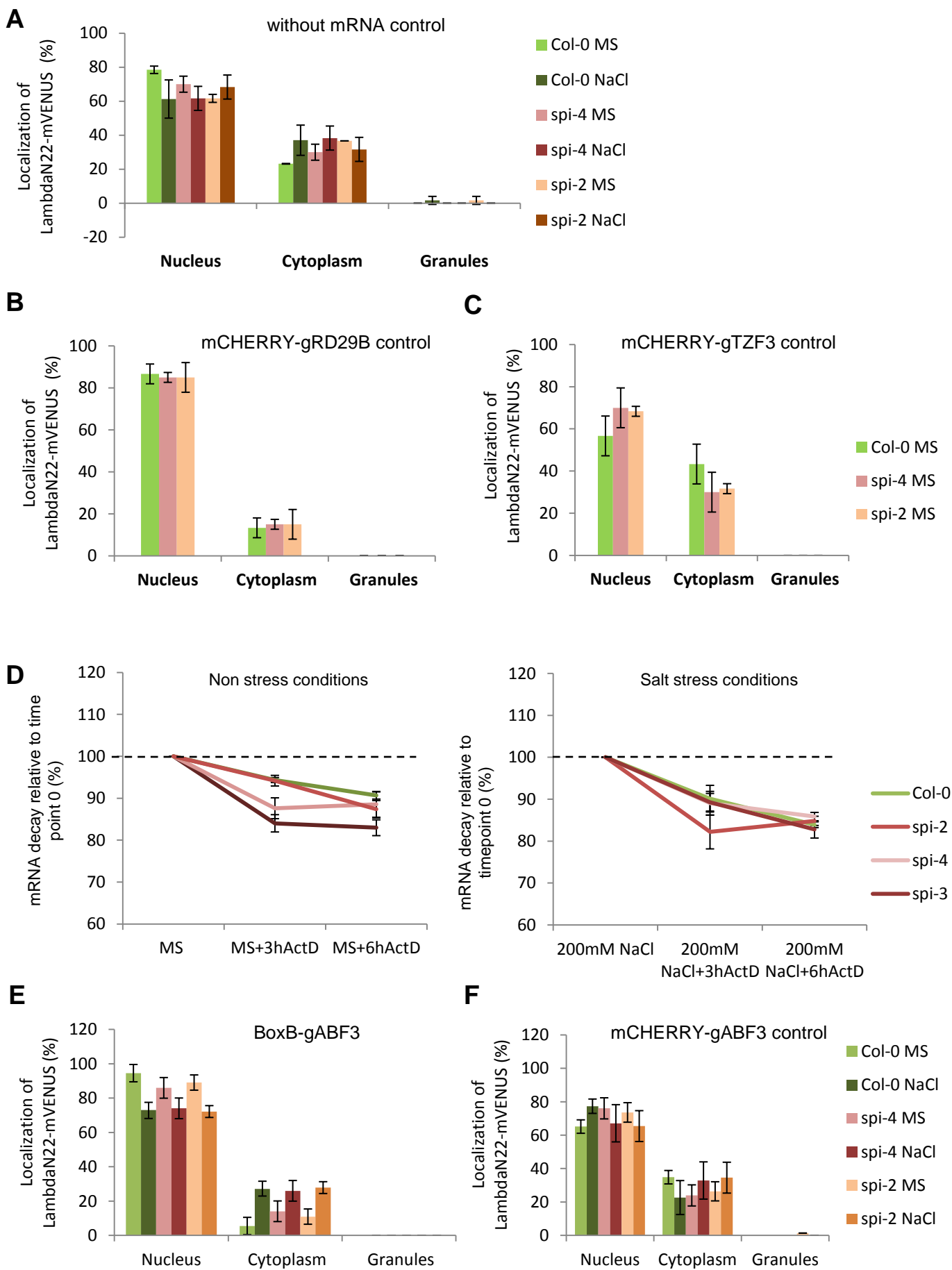

Supplement: S10 Fig — (A) Distribution of LambdaN22-mVENUS reporter coexpressed with the BoxB without any mRNA target under nonstress (½MS) and salt stress (NaCl; ½MS supplemented with 140 mM NaCl for 10 h) conditions in transfected leaf epidermis cells. Data denote the average of 60 cells. Error bars represent standard deviations. Distribution of LambdaN22-mVENUS reporter coexpressed with (B) RD29B mRNA N-terminally fused to mCHERRY (n = 30 cells) and (C) TZF3 mRNA N-terminally fused to mCHERRY (n = 120 cells) under nonstress (½MS) conditions. Error bars represent standard deviations. (D) mRNA stabilities of ABF3 (in %) were determined 3 h and 6 h after application of Actinomycin D (ActD) relative to time point 0 under nonstress (½MS) and salt stress conditions (200 mM NaCl in ½MS liquid medium for 4 h). Data denote the average from three independent biological and two technical replicates. Error bars represent the standard error of the mean. No significant changes between wild-type and spi mutants could be determined (two-tailed student’s t tests). (E) Distribution of LambdaN22-mVENUS reporter coexpressed with gABF3 N-terminally fused to BoxB repeats and with (F) gABF3 N-terminally fused to mCHERRY under nonstress (½MS) and salt stress (NaCl) conditions. (PDF) [file pbio.1002188.s011.pdf]

**Figure S11**

**A**

gRD29B

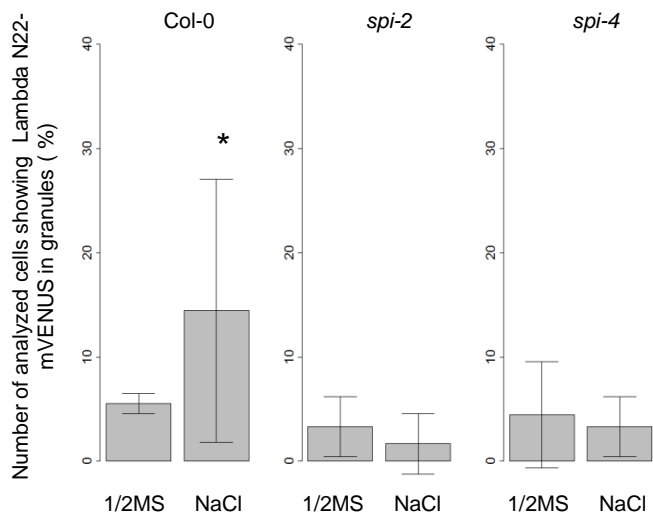

**B**

gTZF3

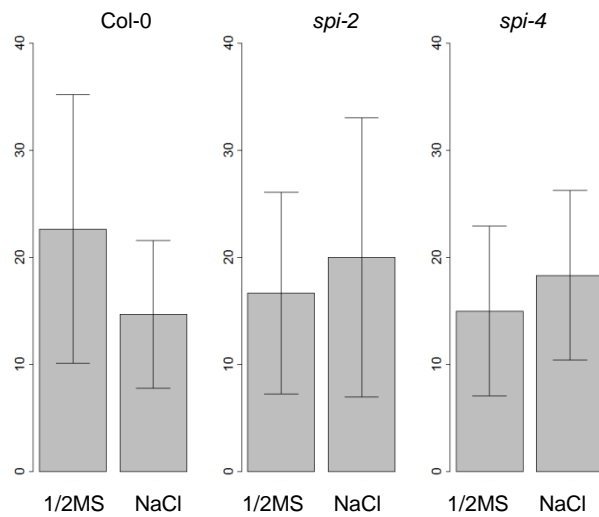

**C**

gTZF3

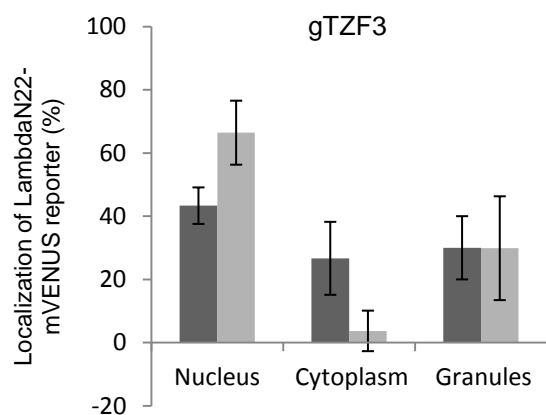

**D**

gRD29B

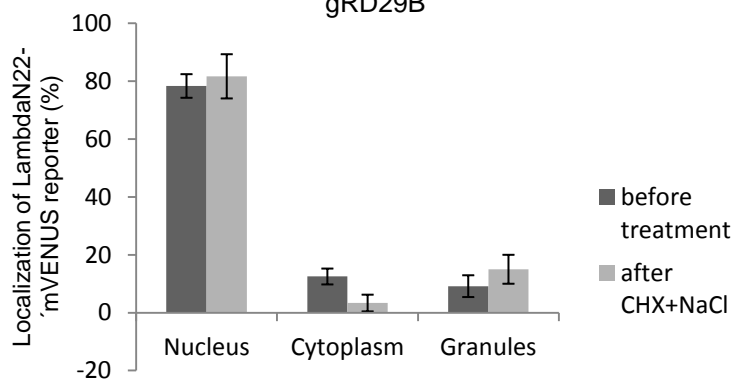

Supplement: S11 Fig — Data denote the average number of cells (in %) showing cytoplasmic accumulations of the LamdaN22-mVENUS reporter in cells coexpressing (A) 16BoxB-gRD29B (n = 65 cells), (B) 16BoxB-gTZF3 (n = 150 cells) under nonstress (-NaCl) and salt stress (NaCl; ½MS supplemented with 140 mM NaCl for 10 h) conditions. Presented are the averages from four biological replicates of RD29B (n = 20 cells each) and seven biological replicates of TZF3 (n = 20 cells each). (C) Distribution of LambdaN22-mVENUS reporter before and after simultaneous treatments with 0.5mM CHX and 140 mM NaCl for 90 min in cells cotransfected with 16BoxB-gRD29B (n = 10 cells per replicate) and (D) 16B-gTZF3 (n = 15 cells per replicate). Presented are the averages from three biological replicates. Error bars represent standard deviations. Two-tailed student’s t tests were performed to compare nonstress and salt stress conditions (* p < 0.05). (PDF) [file pbio.1002188.s012.pdf]

Figure S14

A

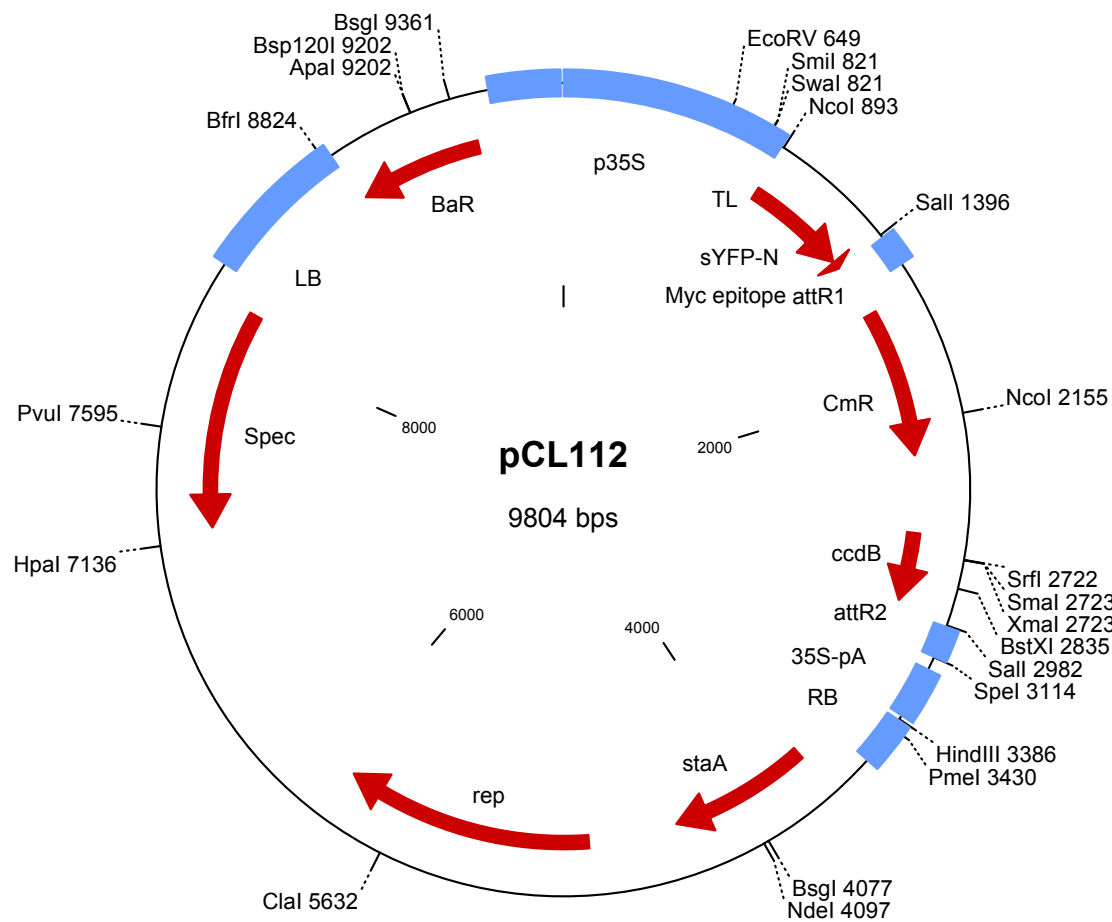

B

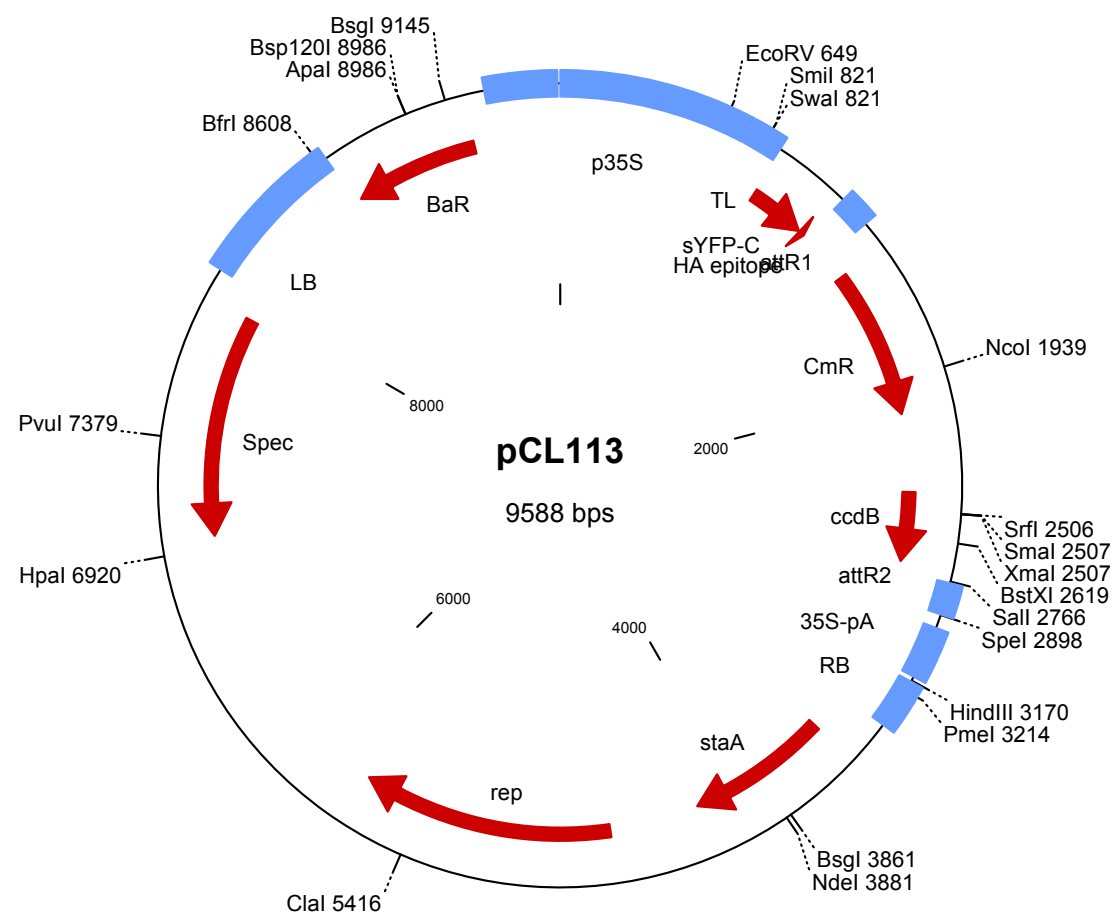

Supplement: S14 Fig — Annotated drawing of Gateway®- compatible A) pCL112- and B) pCL113- vectors used for BiFC assays (donated by Joachim Uhrig). (PDF) [file pbio.1002188.s015.pdf]
