# Supplementary material for: The BEACH Domain Protein SPIRRIG Is Essential for Arabidopsis Salt Stress Tolerance and Functions as a Regulator of Transcript Stabilization and Localization
Source: PLoS Biol. 2015 Jul 2;13(7):e1002188. doi: 10.1371/journal.pbio.1002188 (PMC4489804; doi:10.1371/journal.pbio.1002188)
Supplement: S12 Fig — Residues forming the aromatic triade in the hydrophobic cleft in the EVH1 domain (underlined with a dark grey bar) are in bold. Asterisks highlight positions that have a single, fully conserved residue. Colons indicate conservation between groups of strongly similar properties—scoring >0.5 in the Gonnet PAM 250 matrix. Periods represent conservation between groups of weakly similar properties—scoring <0.5 in the Gonnet PAM 250 matrix. (PDF) [file pbio.1002188.s013.pdf]

Figure S12

```
ScDcp1p      MTGAATAAENSATQLEFYRKALNFNVIGRYDPKIKQLLFHTPHASLYKWDFKKDEWNKLE           60
AtDCP1       MSQ---NGKIIPNLDQNSTRLLNLTVLQRIDPYIEEILITAAHVTFYEFNIELSQWSRKD           57
HsDCP1a      -----------MEALSRAGQEMSLAALKQHDPYITSIADLTGQVALYTFCPKANQWEKTD           49
HsDCP1b      ------MAAVAAGGLVGKGRDISLAALQRHDPYINRIVDVASQVALYTFGHRANEWEKTD           54
                                : :.: .: : ** *  :   : :.::* :  . .:*.: :

ScDcp1p      YQGVLAIYLRDVSQNTNLLPVSPQEVDIFDSQNGSNNIQVNNGSDNSNRNSSGNGNSYKS           120
AtDCP1       VEGSLFVVKRSTQPR---------------------------------------------           72
HsDCP1a      IEGTLFVYRRSASPY---------------------------------------------           64
HsDCP1b      VEGTLFVYTRSASPK---------------------------------------------           69
              :* * :  *...

ScDcp1p      NDSLTYNCGKTLSGKDIYNYGLIILNRINPDNFSMGIVPNSVVNKRKVFNAEEDTLNPLE           180
AtDCP1       -------------------FQFIVMNRRNTDNLVENL---------------------LG           92
HsDCP1a      -------------------HGFTIVNRLNMHNLVEPV---------------------NK           84
HsDCP1b      -------------------HGFTIMNRLSMENRTEPI---------------------TK           89
                                . : ::** . .*    :

ScDcp1p      CMGVEVKDELVIIKNLKHEVYGIWIHTVSDRQNIYELIKYLLENEPKDSFA---------           231
AtDCP1       DFEYEVQGPYLLYRNASQEVNGIWFYNKRECEEVATLFNRILSAYSKVNQKP-KASSS--           149
HsDCP1a      DLEFQLHEPFLLYRNASLSIYSIWFYDKNDCHRIAKLMADVVEEETRRSQQAARDKQSPS           144
HsDCP1b      DLDFQLQDPFLLYRNARLSIYGIWFYDKEECQRIAELMKNLTQYEQLKAHQGTGAGISPV           149
              :  :::   :: :*   .: .**::   : ..:  *:  : .

ScDcp1p      ------------------------------------------------------------           231
AtDCP1       ----KSEFEELEAKPTMAVMDGPLEPSSTARDAPDDPAFVNFFSSTMNLGNTASGSASGP           205
HsDCP1a      QANGCSDHRPIDILEMLSRAKDEYERNQMG----DSNISSP--------GLQPSTQLSNL           192
HsDCP1b      IL-NSGEGKEVDILRMLIKAKDEYTKCKTC----SEPKKIT--------SS--SAIYDNP           194

ScDcp1p      ------------------------------------------------------------           231
AtDCP1       YQSSAIPHQPHQPHQPTIAPPVAAAAPPQIQSPPPLQSSSPLMTLFDNN-----PEV---           257
HsDCP1a      GSTETLEEMPSGSQDKSA---------------PSGHKHLTVEELFGTSLPKEQPAVVGL           237
HsDCP1b      NLIKPIPVKPSENQQQRIPQPNQT--------LDPEPQHLSLTALFGKQDKATCQETVEP           246

ScDcp1p      ------------------------------------------------------------           231
AtDCP1       ------------------------ISSNS----NIHTDLVTPSFFGPPRMM---------           280
HsDCP1a      ----------DSEEMERLPGDA------SQKEPNSFLPFPFEQLGGAPQSETLGVPSAAH           281
HsDCP1b      PQTLHQQQQQQQQQEKLPIRQGVVRSLSYEEPRRHSPPIEKQLC--PAIQKLMVRSADL            304

ScDcp1p      ------------------------------------------------------------           231
AtDCP1       --AQPHLIP-GVSMPTAPPLNPNNASHQ-QRSYGTPVLQPFPPPTPPPSLAPAPT-----           331
HsDCP1a      HSVQPEITTPVLITPASIT----QSNEKHAPTYTIPL-SPVLSPTLPAEAPTAQVPPSLP           336
HsDCP1b      HP--------LSELPENRPCE-NGSTHSAGEFFTGPV-QPG----SPHNIGTSRGVQNAS           350

ScDcp1p      ------------------------------------------------------------           231
AtDCP1       ------------------------------------------------------------           331
HsDCP1a      RNSTMMQAVKTTPR--------------------QRSPL------------------LNQ           358
HsDCP1b      RTQNLFEKLQSTPGAANKCDPSTPAPASSAALNRSRAPTSVTPVAPGKGLAQPPQAYFNG           410

ScDcp1p      ------------------------------------------------------------           231
AtDCP1       ------------------------------------------------------------           331
HsDCP1a      PVPE--LSHASLIANQSPF---RAPLNVTNTA-GTSLPSVDLLQKLRLTPQHDQIQTQPL           412
HsDCP1b      SLPPQTVGHQAHGREQSTLPRQTLPISGSQTGSSGVISPQELLKKLQIVQQEQQLHAS--           468

ScDcp1p      ------------------------------------------------------------           231
AtDCP1       ------------------------------------------------------------           331
HsDCP1a      GKGAMVASFSP---AAGQLATPESFIEPPSKTAAARVAASASLSNMVLAPLQSMQQNQDP           469
HsDCP1b      NRPALAAKFPVLAQSSGTGKPLESWINKTPNTE-----------------------QQT           504

ScDcp1p      ------------------------------------------------------------           231
AtDCP1       ------------------------------------------------------------           331
HsDCP1a      EVFVQPKVLSSAIQVAGAPLVTATTTAVSSVLLAPSVFQQTVTRSSD-----LE------           518
HsDCP1b      PLF----------QVISP--QRIPATAAPSLLMSPMVFAQPTSVPPKERESGLLPVGGQE           552

ScDcp1p      -----------------------------------------------------------------      231
AtDCP1       ---------------------GPVISRDKVKEALLSLLQEDE-FIDKITRTLQNALQQ-------      367
HsDCP1a      -RKASSPSPLTIGTPESQRKPSIILSKSQLQDTLIHLIKNDSSFLSTLHEVYLQVLTKNKDNHNL      582
HsDCP1b      PPAAATSLLLPIQSPEPSVITSSPLTKLQLQEALLYLIQNDDNFLNIIYEAYLFSMTQAAMKKTM      617
                                                 *   *  *
```
