# Supplementary material for: The BEACH Domain Protein SPIRRIG Is Essential for Arabidopsis Salt Stress Tolerance and Functions as a Regulator of Transcript Stabilization and Localization
Source: PLoS Biol. 2015 Jul 2;13(7):e1002188. doi: 10.1371/journal.pbio.1002188 (PMC4489804; doi:10.1371/journal.pbio.1002188)
Supplement: S13 Fig — The GRAM and PH domains of FAN and SPI are shaded in dark and light gray, respectively. The BEACH domain in both proteins is underlined. The region containing WD40 repeats is printed in bold; the part that is not included in the clone used in this study is in italic letters. Asterisks highlight positions that have a single, fully conserved residue. Colons indicate conservation between groups of strongly similar properties—scoring >0.5 in the Gonnet PAM 250 matrix. Periods represent conservation between groups of weakly similar properties—scoring <0.5 in the Gonnet PAM 250 matrix. (PDF) [file pbio.1002188.s014.pdf]

Figure S13

```
FAN      ------------------------------------------------------------    0
SPI      MKWATLLKGTEDPEWQLCPIEGPYRMRKKLERCKLKIDSIQNVLDGKLELGEIELPKVKN    60


FAN      ------------------------------------------------------------    0
SPI      EDGPVISDTDSEPPFLLSELYDESFLKESDDFKDVASARNGWNDDRASSTNEASLHSALD    120


FAN      ------------------------------------------------------------    0
SPI      FGGKSSIASVPITDTTHVKSETGSPRHSSSAKMDETNGREEKSEKELNDDGEYLIRPYLE    180


FAN      SFDKNRFQNI--------------------------------SEKLHMECKAEM------    22
SPI      HLEKIRFRYNCERVVDLDKHDGIFLIGEFCLYVIENFYIDEDGCICEKECEDELSVIDQA    240
          ::* **:                                 .   . **: *:

FAN      ---------------------VTPLV---------------TNPGHVCITDTNLYFQPLN    46
SPI      LGVKKDVSGSSDFHSKSSTSWTTTVKTGAVGGRAWAYGGGAWGKEKMCM---------TG    291
                              .* :                    ::*:

FAN      GYPKPVVQITLQDVRRIYKRRHGLMPLGLEVFCTEDDLCSDIYLKFYEPQDRDDLYFYIA    106
SPI      NLPHPWRMWKLNNVHEILKRDYQLRPVAIEIFSMD--GCNDLLV--FHKKEREEVFKNLV    347
           *:*    .*::*:.* ** : * *:.:*:*. :   *.*: :  :. ::*::::  :.

FAN      T-------YL--------------EHHVAEHTAESYMLQWQRGHLSNYQYLLHLNNLADR    145
SPI      AMNLPRNSMLDTTISGSAKQESNEGGRLFKLMAKSFSKRWQNGEISNFQYLMHLNTLAGR    407
         :        *               :: :  *:*:  :**.*.:**:***:***.** *

FAN      SCNDLSQYPVFPWIIHDYSSSELDLSNPGTFRDLSKPVGALNKERLERLLTRYQ---EMP    202
SPI      GYSDLTQYPVFPWVLADYDSESLDFSDPKTFRKLHKPMGCQTPEGEEEFRKRYESWDDPE    467
         . .**:*******:: **.*..**:*:* ***.* **:*. . *  *.: .**:   :

FAN      EPKFMYGSHYSSPGYVLFYLVRIAPEY--MLCLQNGRFDNADRMFNSIAETWKNCLD--G    258
SPI      VPKFHYGSHYSSAGIVLFYLIRLPPFSSENQKLQGGQFDHADRLFNSIKDTWLSAAGKGN    527
          *** ******* * *****:*: *       ** *:**.***:**** :** ..

FAN      ATDFKELIPEFYGDDVSFLVNSLKLDLGKRQGGQMVDDVELPPWASS-PEDFLQKSKDAL    317
SPI      TSDVKELIPEFFYM-PEFLENRFSLDLGEKQSGEKVGDVFLPPWARGSVREFILKHREAL    586
         ::*.*******:    .** * :.****::*.*: * ** *****  .  .:*: * ::**

FAN      ESNYVSEHLHEWIDLIFGYKQKGSDAVGAHNVFHPLTYEGGVDLNSIQDPDEKVAMLTQI    377
SPI      ESDYVSENLHHWIDLIFGYKQRGKAAEEAVNVFYHYTYEGNVDIDAVTDPAMKASILAQI    646
         **:****.**.**********:*.  *  * ***:  **** **:::: **  *.::*:**

FAN      LEFGQTPKQLFVTPHPRRITPKFKSLSQTSSYNASMADSPGEESFEDLTEESKTLAWNNI    437
SPI      NHFGQTPKQLFPKAHVKRRTDRKIPLHPLK---HSMHLVPHEI---------RKCS-SSI    693
          .********* . * :* * :   *   .    **   * *          :. : ..*

FAN      T-KLQLHEHYKIHKEAVTGITVSRNGSSVFTTSQDSTLKMFSKESKMLQRSISFSNMALS    496
SPI      SQIITFHDKV------------L---------------------------------VAGA    708
         :  : :*::                                               :* :

FAN      SCLLLPGDATVITSSWDN---NVYFYSIAFGRRQDTLMGHDDA-------VSKICWHDNR    546
SPI      NCFLKPRGYTKY-ITWGFPDRSLRFMSY----DQDKLLSTHENLHESNQIQCAGVSHDGR    763
         .*:* *   *    :*     .: * *      **.*:. .:         .    ** *

FAN      L-YSASWDSTVKVWSGVPAEMPGTKRHHFDLLAELEHDVSVDTISLNAASTLLVSGTKEG    605
SPI      IVVTGAEDGLVCVWRVSKDGPRGSRRLRLEK-ALCAHTAKVTCLRVSQPYMMIASGSDDC    822
         :  :.: *. * **        *::* :::  *   * ..*  : :.    ::.**:.:

FAN      TVNIWDLTTATLMHQIPCHSGIVCDTAFSPDSRHVLSTG----------TDGCLNVIDVQ    655
SPI      TVIIWDLSSLSFVRQLPDFPVPISAIYIN-DLTGEIVTAAGTVLAVWSINGDCLAVANTS    881
         ** ****:: ::::*:* .   :.   :. *    : *.          .  ** * :..

FAN      IGMLISSMTSDEPQRCFVW-DGNSVLSGSQSGELLVWDLLGAKISERIQ-----------    703
SPI      QL-PSDSVLSVTGSTSSDWLETSWYVTGHQSGAVKVWRMIHCTDPVSAESKTSSSNRTGG    940
              .*: *   . .  * : .  ::* *** : ** :: ..     :

FAN      -------------------GHTGAVTCIWMNEQCSSIITGGEDRQIIFWKLQY-------    737
SPI      LNLGDQVPEYKLILHKVLKFHKQPVTALHLTSDLKQLLSGDSAGQLLSWTVPDETLRASM    1000
                            *.  **.: :..: ..:::* .  *:: *.:

FAN      --------------------- 737
SPI      KQASLKQASLKQASLKQASSV 1021
```
