# Supplementary material for: The BEACH Domain Protein SPIRRIG Is Essential for Arabidopsis Salt Stress Tolerance and Functions as a Regulator of Transcript Stabilization and Localization
Source: PLoS Biol. 2015 Jul 2;13(7):e1002188. doi: 10.1371/journal.pbio.1002188 (PMC4489804; doi:10.1371/journal.pbio.1002188)
Supplement: S1 Table — Total numbers of analyzed cells are provided. (DOCX) [file pbio.1002188.s016.docx]

**S1 Table.** Quantification of BiFC assays in cells co-expressing DCP2-mCHERRY. Total numbers of analyzed cells are provided.

| Co-expression of  DCP2-mCHERRY | YFP_C_-DCP1 and YFP_N_-SPI-PBW | YFP_C-_AtMYC1- and YFP_N_-SPI-PBW | VPS20.2-YFP_N_ and YFP_C_-DCP1 |
| --- | --- | --- | --- |
| Total number of analyzed cells (derived from 3 biol. replicates) | 90 | 90 | 90 |
| Cytoplasmic signal | 4 | 0 | 0 |
| Cytoplasmic dots | 86 | 0 | 0 |
