# Supplementary material for: The BEACH Domain Protein SPIRRIG Is Essential for Arabidopsis Salt Stress Tolerance and Functions as a Regulator of Transcript Stabilization and Localization
Source: PLoS Biol. 2015 Jul 2;13(7):e1002188. doi: 10.1371/journal.pbio.1002188 (PMC4489804; doi:10.1371/journal.pbio.1002188)
Supplement: S2 Table — Total numbers of analyzed cells are provided. (DOCX) [file pbio.1002188.s017.docx]

**S2 Table.** Quantification of BiFC assays in cells co-expressing free RFP. Total numbers of analyzed cells are provided.

| Co-expression of  free RFP | YFP_C_-DCP1 and YFP_N_-SPI-PBW | YFP_C_-AtMYC1 and YFP_N_-SPI-PBW | YFP_N_-VPS20.2 and YFP_C_-DCP1 |
| --- | --- | --- | --- |
| Total number of analyzed cells (derived by 3 biol. replicates) | 90 | 90 | 90 |
| Cytoplasmic signal | 17 | 0 | 0 |
| Cytoplasmic dots | 73 | 0 | 0 |
