# Supplementary material for: The BEACH Domain Protein SPIRRIG Is Essential for Arabidopsis Salt Stress Tolerance and Functions as a Regulator of Transcript Stabilization and Localization
Source: PLoS Biol. 2015 Jul 2;13(7):e1002188. doi: 10.1371/journal.pbio.1002188 (PMC4489804; doi:10.1371/journal.pbio.1002188)
Supplement: S3 Table — Differentially enriched transcripts in Col-0 and spi are highlighted by black boxes. (DOCX) [file pbio.1002188.s018.docx]

**S3 Table.** Comparison of hyperosmotic salinity and salt stress response GO-Term categories*.* Differentially enriched transcripts in Col-0 and *spi* are highlighted by black boxes.

| **GO Term** | **hyperosmotic salinity** | **hyperosmotic salinity** | **salt stress** | **salt stress** |
| --- | --- | --- | --- | --- |
| Genotype | Col-0 up | *sp*i up | Col-0 up | *sp*i up |
|  | AT1G06620 | AT1G06620 | AT1G03220 | AT1G61210 |
|  | AT1G15430 | AT1G15430 | AT1G01520 | AT1G01520 |
|  | AT1G73480 | AT1G63720 | AT1G06620 | AT1G06620 |
|  | AT1G80160 | AT1G73480 | AT1G15430 | AT1G13930 |
|  | AT2G05940 | AT1G80160 | AT1G16850 | AT1G15430 |
|  | AT2G27310 | AT2G05940 | AT1G23190 | AT1G16850 |
|  | AT2G38905 | AT2G27310 | AT1G55450 | AT1G23190 |
|  | AT2G43320 | AT2G38905 | AT1G61210 | AT1G29660 |
|  | AT3G05640 | AT2G43320 | AT1G61340 | AT1G61340 |
|  | AT3G19970 | AT3G05640 | AT1G61890 | AT1G61890 |
|  | AT3G20300 | AT3G19970 | AT1G62660 | AT1G62660 |
|  | AT4G18950 | AT3G20300 | AT1G63010 | AT1G63010 |
|  | AT4G30650 | AT4G30650 | AT1G63720 | AT1G63720 |
|  | AT4G30660 | AT4G30660 | AT1G73480 | AT1G73480 |
|  | AT5G22540 | AT5G22540 | AT1G74840 | AT1G80160 |
|  | AT5G67340 | AT5G67340 | AT1G80160 | AT2G05940 |
|  |  |  | AT2G05940 | AT2G20880 |
|  |  |  | AT2G20880 | AT2G23840 |
|  |  |  | AT2G27310 | AT2G27310 |
|  |  |  | AT2G33700 | AT2G33700 |
|  |  |  | AT2G37760 | AT2G37760 |
|  |  |  | AT2G37770 | AT2G37770 |
|  |  |  | AT2G38340 | AT2G38340 |
|  |  |  | AT2G38905 | AT2G38905 |
|  |  |  | AT2G43320 | AT2G43320 |
|  |  |  | AT2G44060 | AT2G44060 |
|  |  |  | AT3G05640 | AT3G05640 |
|  |  |  | AT3G09600 | AT3G09200 |
|  |  |  | AT3G12320 | AT3G09600 |
|  |  |  | AT3G19970 | AT3G11250 |
|  |  |  | AT3G20300 | AT3G12320 |
|  |  |  | AT3G20390 | AT3G19970 |
|  |  |  | AT4G01280 | AT3G20300 |
|  |  |  | AT4G11890 | AT3G27240 |
|  |  |  | AT4G15430 | AT3G53870 |
|  |  |  | AT4G15920 | AT3G60750 |
|  |  |  | AT4G18950 | AT4G01280 |
|  |  |  | AT4G20380 | AT4G04330 |
|  |  |  | AT4G23670 | AT4G11890 |
|  |  |  | AT4G24800 | AT4G15430 |
|  |  |  | AT4G30650 | AT4G15920 |
|  |  |  | AT4G30660 | AT4G23670 |
|  |  |  | AT4G33940 | AT4G24800 |
|  |  |  | AT4G38360 | AT4G30650 |
|  |  |  | AT5G02020 | AT4G30660 |
|  |  |  | AT5G06980 | AT4G38360 |
|  |  |  | AT5G20700 | AT5G02020 |
|  |  |  | AT5G22540 | AT5G06980 |
|  |  |  | AT5G38895 | AT5G20080 |
|  |  |  | AT5G52660 | AT5G20700 |
|  |  |  | AT5G67340 | AT5G22540 |
|  |  |  |  | AT5G38895 |
|  |  |  |  | AT5G52660 |
|  |  |  |  | AT5G62350 |
|  |  |  |  | AT5G67340 |
|  |  |  |  |  |
