# Supplementary material for: The BEACH Domain Protein SPIRRIG Is Essential for Arabidopsis Salt Stress Tolerance and Functions as a Regulator of Transcript Stabilization and Localization
Source: PLoS Biol. 2015 Jul 2;13(7):e1002188. doi: 10.1371/journal.pbio.1002188 (PMC4489804; doi:10.1371/journal.pbio.1002188)
Supplement: S4 Table — Displayed are the mean expression values and for 19 salt stress-responsive genes in Col-0 (wt) and spi (mut) mutants under nonstress and salt stress (NaCl) conditions. Expression changes between control and salt stress (NaCl) conditions as well as Col-0 and spi are presented as log-fold change. Statistically significant changes between spi and Col-0 are highlighted by red boxes (q-values, BH-corrected). (DOCX) [file pbio.1002188.s019.docx]

**S4 Table.** Gene expression pattern of selected salt stress-regulated transcripts. Displayed are the mean expression values of 19 salt stress-responsive genes in Col-0 (wt) and *spi* (mut) under non-stress and salt-stress (NaCl) conditions. Expression changes between control and salt stress conditions as well as Col-0 and *spi* are presented as log-fold change. Statistically significant changes between *spi* and Col-0 are highlighted by red boxes (q-values, BH-corrected).

|  |  |  | Average of gene expression | | |  | log-fold change | |  |  | q-values | |  |  |
| --- | --- | --- | --- | --- | --- | --- | --- | --- | --- | --- | --- | --- | --- | --- |
| **ATG-Number** | **abbreviation** | **name** | **Average wt non-stress** | **Average mutant non-stress** | **Average wt+NaCl** | **Average mut+NaCl** | **log (mut/wt) non-stress** | **log (wtNacl/wt) non-stress** | **log (mutNaCl/mut)** | **log (mutNaCl/wtNaCl)** | **wt vs mut non-stress** | **wt vt wt NaCl** | **mut vs mut NaCl** | **wtNaCl vs mut NaCl** |
| **AT2G01980** | SOS1 | SALT OVERLY SENSITIVE 1 | 15,78 | 15,10 | 15,02 | 17,17 | -0,06 | -0,07 | 0,17 | 0,18 | 1,000 | 0,821 | 0,235 | 0,426 |
| **AT5G35410** | SOS2 | SALT OVERLY SENSITIVE 2 | 8,04 | 9,58 | 8,36 | 8,95 | 0,23 | 0,05 | -0,09 | 0,09 | 1,000 | 1,000 | 1,000 | 0,896 |
| **AT5G24270** | SOS3 | SALT OVERLY SENSITIVE 3 | 21,35 | 25,20 | 10,86 | 11,42 | 0,23 | -0,91 | -1,08 | 0,07 | 1,000 | 0,000 | 0,000 | 0,994 |
| **AT1G01140** | CIPK9 | CBL-INTERACTING PROTEIN KINASE 9 | 98,12 | 85,11 | 157,40 | 163,58 | -0,20 | 0,68 | 0,93 | 0,06 | 0,544 | 0,000 | 0,000 | 0,908 |
|  |  |  |  |  |  |  |  |  |  |  |  |  |  |  |
| **At4g29190** | TZF3 | TANDEM ZINC FINGER PROTEIN 3 | 45,55 | 49,85 | 63,45 | 52,85 | 0,13 | 0,47 | 0,08 | -0,26 | 1,000 | 0,001 | 0,560 | 0,270 |
| **AT3G55980** | TZF11 | Tandem Zinc Finger Protein 11 | 26,53 | 33,38 | 118,76 | 100,05 | 0,32 | 2,12 | 1,56 | -0,25 | 0,666 | 0,000 | 0,000 | 0,693 |
| **AT4G26080** | ABI1 | ABA INSENSITIVE 1 | 93,66 | 96,34 | 629,64 | 468,63 | 0,04 | 2,74 | 2,27 | -0,43 | 1,000 | 0,000 | 0,000 | 0,004 |
| **AT5G57050** | ABI2 | ABA INSENSITIVE 2 | 4,87 | 6,19 | 88,77 | 63,80 | 0,29 | 3,94 | 3,17 | -0,47 | 0,996 | 0,000 | 0,000 | 0,009 |
| **AT4G34000** | ABF3 | BSCISIC ACID RESPONSIVE ELEMENTS-BINDING FACTOR 3 | 50,52 | 69,26 | 518,79 | 401,25 | 0,45 | 3,33 | 2,52 | -0,37 | 0,113 | 0,000 | 0,000 | 0,033 |
| **AT5G52300** | RD29B | RESPONSIVE TO DESSICATION 29B | 2,01 | 3,68 | 236,95 | 180,91 | 0,64 | 6,31 | 5,28 | -0,39 | 0,562 | 0,000 | 0,000 | 0,398 |
| **AT5G52310** | RD29A | RESPONSIVE TO DESSICATION 29A | 140,02 | 77,26 | 2373,37 | 1686,73 | -0,85 | 4,07 | 4,43 | -0,49 | 0,000 | 0,000 | 0,000 | 0,003 |
| **AT3G11020** | DREB2B | DEHYDRATION-RESPONSIVE ELEMENT BINDING PROTEIN 2 | 3,03 | 4,40 | 29,41 | 28,92 | 0,42 | 2,92 | 2,47 | -0,02 | 0,799 | 0,000 | 0,000 | 1,000 |
| **AT4G25490** | DREB1B | dehydration responsive element-binding factor 1B | 0,08 | 0,26 | 22,23 | 20,46 | 0,22 | 4,42 | 4,09 | -0,11 | 0,992 | 0,000 | 0,000 | 0,995 |
| **AT4G25470** | DREB1C | dehydration responsive element-binding factor 1C | 0,95 | 1,21 | 24,88 | 26,76 | 0,18 | 3,73 | 3,65 | 0,10 | 1,000 | 0,000 | 0,000 | 0,712 |
| **AT3G19290** | ABF4 | BSCISIC ACID RESPONSIVE ELEMENTS-BINDING FACTOR 4 | 76,62 | 81,16 | 157,43 | 132,32 | 0,08 | 1,03 | 0,70 | -0,25 | 1,000 | 0,000 | 0,000 | 0,340 |
| **AT1G45249** | ABF2 | BSCISIC ACID RESPONSIVE ELEMENTS-BINDING FACTOR 2 | 18,92 | 17,37 | 68,95 | 55,09 | -0,12 | 1,81 | 1,61 | -0,32 | 1,000 | 0,000 | 0,000 | 0,147 |
| **AT1G56010** | NAC1 | NAC DOMAIN CONTAINING PROTEIN 21 | 18,30 | 22,38 | 15,01 | 19,45 | 0,28 | -0,27 | -0,19 | 0,35 | 0,980 | 0,102 | 0,722 | 0,173 |
| **AT3G15510** | NAC2 | NAC DOMAIN CONTAINING PROTEIN 2 | 12,65 | 12,29 | 8,75 | 7,53 | -0,04 | -0,49 | -0,64 | -0,19 | 1,000 | 0,009 | 0,003 | 0,868 |
