# Supplementary material for: The BEACH Domain Protein SPIRRIG Is Essential for Arabidopsis Salt Stress Tolerance and Functions as a Regulator of Transcript Stabilization and Localization
Source: PLoS Biol. 2015 Jul 2;13(7):e1002188. doi: 10.1371/journal.pbio.1002188 (PMC4489804; doi:10.1371/journal.pbio.1002188)
Supplement: S5 Table — (DOCX) [file pbio.1002188.s020.docx]

**S5 Table.** Sequences of primers used for cloning.

| **Primer** | **Sequence (5´to 3´)** |
| --- | --- |
| SPI-PBW fw | GGGGACAAGTTTGTACAAAAAAGCAGGCTCAATGAAATGGGCAACATTGCTTAAGGGGACTGAA |
| SPI-PBW rev | GGGGACCACTTTGTACAAGAAAGCTGGGTATWAAACCGATGAAGCCTGTTTCAGTGAAGC |
| DCP1 fw | GGGACAAGTTTGTACAAAAAAGCAGGCTTAATGTCTCAAAACGGGAAGAT |
| DCP1 rev | GGGGACCACTTTGTACAAGAAAGCTGGGTTTWATTGTTGAAGTGCATTTT |
| DCP5 fw | GGGACAAGTTTGTACAAAAAAGCAGGCTTAATGGCGGCTGATAATACGGG |
| DCP5 rev | GGGGACCACTTTGTACAAGAAAGCTGGGTTTWAGGTAGTACGATTTGATA |
| VCS fw | GGGGACAAGTTTGTACAAAAAAGCAGGCTTAATGGCGTCTTCTCCTGGTAA |
| VCS rev | GGGGACCACTTTGTACAAGAAAGCTGGGTTTWATTTGCAACCCATAAGCA |
| DCP2 fw | GGGGACAAGTTTGTACAAAAAAGCAGGCTTAATGTCGGGCCTCCATCGATC |
| DCP2 rev | GGGGACCACTTTGTACAAGAAAGCTGGGTTTCCAGCTGAATTACCAGATT |
| DCP1a fw | GGGGACAAGTTTGTACAAAAAAGCAGGCTTAATGGAGGCGCTGAGTCGAGC |
| DCP1a rev | GGGGACCACTTTGTACAAGAAAGCTGGGTTTWATAGGTTGTGGTTGTCTT |
| DCP1b fw | GGGGACAAGTTTGTACAAAAAAGCAGGCTTAATGGCAGCCGTGGCGGCAGG |
| DCP1b rev | GGGGACCACTTTGTACAAGAAAGCTGGGTTTWACATAGTCTTTTTCATGG |
| DCP1p fw | GGGGACAAGTTTGTACAAAAAAGCAGGCTTAATGACCGGAGCAGCAACTGC |
| DCP1p rev | GGGGACCACTTTGTACAAGAAAGCTGGGTTTWAAGCAAAAGAATCTTTTG |
| FAN-PB fw | GGGGACAAGTTTGTACAAAAAAGCAGGCTTACTCGAGATGTCATTTGACAAAAACAGGTT |
| FAN-PB rev | GGGGACCACTTTGTACAAGAAAGCTGGGTGTCGACCTWACTTTGGGGTGATCCTTCGA |
| gRD29B fw | GGGGACAAGTTTGTACAAAAAAGCAGGCTTAAGATCTAAGCAACAATCAGA |
| gRD29B rev | GGGGACCACTTTGTACAAGAAAGCTGGGTTATCCAAAAAAGAAATATTTT |
| gTZF3 fw | GGGGACAAGTTTGTACAAAAAAGCAGGCTTAAACACAAACAAAAAAAAAGA |
| gTZF3 rev | GGGGACCACTTTGTACAAGAAAGCTGGGTTAGATAATAATTTGCATTCAT |
| gABF3 fw | GGGGACAAGTTTGTACAAAAAAGCAGGCTTAGGTTTGATGAATCGATTTTT |
| gABF3 rev | GGGGACCACTTTGTACAAGAAAGCTGGGTTTAACCGTTTGAAAGCATCTT |
