# Supplementary material for: The BEACH Domain Protein SPIRRIG Is Essential for Arabidopsis Salt Stress Tolerance and Functions as a Regulator of Transcript Stabilization and Localization
Source: PLoS Biol. 2015 Jul 2;13(7):e1002188. doi: 10.1371/journal.pbio.1002188 (PMC4489804; doi:10.1371/journal.pbio.1002188)
Supplement: S6 Table — (DOCX) [file pbio.1002188.s021.docx]

**S6 Table.** Sequences of primers used for qPCR analysis.

| **Primer** | **Sequence (5´to 3´)** |
| --- | --- |
| RD29B q-fw | GAAGAGTCTCCACAATCACTTGG |
| RD29B q-rev | CAACTCACTTCCACCGGAAT |
| CIPK9 q-fw | TACGTTGCCCCTGAGGTT |
| CIPK9 q-rev | AGAATGACACCACAAGACCAGA |
| ABI1 q-fw | CGCTAACTGCGGTGACTCTA |
| ABI1 q-rev | CAATCCTCGCAGCTTCATCT |
| ABF3 q-fw | TGGAAAAGCAGAAAAATCAGC |
| ABF3 q-rev | CAAGCATTGCCTTTTGCAT |
| 18S rRNA fw | AAACGGCTACCACATCCAAG |
| 18S rRNA rev | GACTCGAAAGAGCCCGGTAT |
| TZF3 q-fw | AAGGAGAATTGCATGGATCG |
| TZF3 q-rev | AGGAGCCTCACCCAAATTCT |
