# Supplementary material for: The BEACH Domain Protein SPIRRIG Is Essential for Arabidopsis Salt Stress Tolerance and Functions as a Regulator of Transcript Stabilization and Localization
Source: PLoS Biol. 2015 Jul 2;13(7):e1002188. doi: 10.1371/journal.pbio.1002188 (PMC4489804; doi:10.1371/journal.pbio.1002188)
Supplement: S7 Table — Presented are the numbers of total fragments, mapped fragments, uniquely mapped fragments, nonspecific mappings, unmapped fragments, mapped exons, spanned exon–exon borders, total exons, annotated introns, and the number of sequenced genes per sample. (DOCX) [file pbio.1002188.s022.docx]

**S7 Table.** Additional information on RNA-Seq analysis. Presented are the numbers of total fragments, mapped fragments, uniquely mapped fragments, non-specific mappings, unmapped fragments, mapped exons, spanned exon-exon borders, total exons, annotated introns and the number of sequenced genes per sample.

|  | Col-0 no-stress; replicate 1 | Col-0 no-stress; replicate 2 | *spi* mutant no-stress; replicate 1 | *spi* mutant no-stress; replicate 2 | Col-0 salt-stress; replicate 1 | Col-0 salt-stress; replicate 2 | Col-0 salt-stress; replicate 3 | *spi* mutant salt-stress; replicate 1 | *spi* mutant salt-stress; replicate 2 | *spi* mutant salt-stress; replicate 3 |
| --- | --- | --- | --- | --- | --- | --- | --- | --- | --- | --- |
| total fragments | 19735663 | 18625399 | 16897228 | 14994537 | 18266093 | 14989688 | 18284125 | 12519422 | 15846810 | 14051953 |
| mapped fragments | 19188534 | 17763538 | 16283466 | 14185023 | 17537988 | 14230013 | 17652863 | 11865743 | 15255450 | 13585714 |
| mapped fragments [%] | 97,2 | 95,4 | 96,4 | 94,6 | 96,0 | 94,9 | 96,5 | 94,8 | 96,3 | 96,7 |
| uniquely mapped fragments | 18589459 | 16640818 | 15631045 | 13336150 | 16714739 | 13508435 | 16957159 | 11220036 | 14746364 | 13155874 |
| non-specific mappings | 599075 | 1122720 | 652421 | 848873 | 823249 | 721578 | 695704 | 645707 | 509086 | 429840 |
| unmapped fragments | 547129 | 861861 | 613762 | 809514 | 728105 | 759675 | 631262 | 653679 | 591360 | 466239 |
| exon | 12553336 | 11242638 | 10684340 | 9239386 | 11367440 | 9268585 | 11212815 | 7693217 | 9891913 | 8805365 |
| exon-exon border spanning | 5780284 | 5126747 | 4680910 | 3840144 | 5101067 | 4026892 | 5484270 | 3369343 | 4641129 | 4171341 |
| total exon | 18333620 | 16369385 | 15365250 | 13079530 | 16468507 | 13295477 | 16697085 | 11062560 | 14533042 | 12976706 |
| annotated intron | 255839 | 271433 | 265795 | 256620 | 246232 | 212958 | 260074 | 157476 | 213322 | 179168 |
| total gene | 18589459 | 16640818 | 15631045 | 13336150 | 16714739 | 13508435 | 16957159 | 11220036 | 14746364 | 13155874 |
