# Supplementary material for: The BEACH Domain Protein SPIRRIG Is Essential for Arabidopsis Salt Stress Tolerance and Functions as a Regulator of Transcript Stabilization and Localization
Source: PLoS Biol. 2015 Jul 2;13(7):e1002188. doi: 10.1371/journal.pbio.1002188 (PMC4489804; doi:10.1371/journal.pbio.1002188)
Supplement: S1 Text — General transcriptional changes between Col-0 and spi under nonstress conditions and salt stress-dependent transcriptional changes in Col-0 and spi are presented. (DOCX) [file pbio.1002188.s024.docx]

**S1 Text**

**RNA-Seq Analysis of Col-0 and *spi* mutants**

The transcriptome comparison between Col-0 and *spi* mutants under control and salt stress conditions highlighted the presence of large environmental and clearly discernable genotype effects. The environmental and genotype effects were reflected in the principle component analysis (S3A Fig). Here, the treatment effect is described in the first dimension (76.2% of the variation explained); and the genotype effect in the second dimension (6.5% of the variation explained). Under control conditions *spi* mutants displayed a significantly lower abundance of genes required for the central carbon metabolism in comparison to Col-0 (light reaction q<10^-18^, Calvin Benson Bassham cycle q<10^-6^, tetrapyrrole synthesis q<0.002, S3B; S4 and S5 Fig).

**Salt stress-dependent transcriptional changes in Col-0**

In Col-0, the salt treatment altered the transcriptional abundance of 8469 genes. Enrichments were found for genes required for pathogen response (chitin q<10^-28^, jasmonate q<10^-11^, and callose deposition q<10^-13^), salt stress response (water deprivation q<10^-16^, water q<10^-16^), and stress-related hormones such as ethylene (response q<10^-6^, synthesis q<10^-11^) (Fig6A and B). Additional stress-related categories were enriched to a minor degree. At the same time, genes encoding transporters of ions such as nitrate and iron were of lower abundance (q<10^-26^) as were those involved in the responses to nitrate (q<10^-16^) and iron (q<10^-7^) (S5 and S6 Fig). Summarized, this transcript abundance pattern reflected a cross activation of different stress pathways.

**Salt stress-dependent transcriptional changes in *spi***

The application of salt stress resulted in the same degree of gene category enrichments in *spi* than in Col-0 (Fig6C and D, S7 and 8 Fig). The general salt stress responsiveness of *spi* mutants was confirmed in a direct comparison of gene enrichments in GO (Gene Ontology)-Terms, like “hyperosmotic salinity” (GO:0042538) and “salt stress” (GO:0009651). In both, more than 80% of members were indistinguishable between salt-treated wild type plants and *spi* mutants (S3 Table).
